# Blocking endothelial apoptosis revascularizes the retina in a model of ischemic retinopathy

**DOI:** 10.1172/JCI127668

**Published:** 2020-07-13

**Authors:** Zoe L. Grant, Lachlan Whitehead, Vickie H.Y. Wong, Zheng He, Richard Y. Yan, Abigail R. Miles, Andrew V. Benest, David O. Bates, Claudia Prahst, Katie Bentley, Bang V. Bui, Robert C.A. Symons, Leigh Coultas

**Affiliations:** 1 Walter and Eliza Hall Institute of Medical Research, Parkville, Victoria, Australia.; 2 Department of Medical Biology, and; 3 Department of Optometry and Vision Sciences, University of Melbourne, Parkville, Victoria, Australia.; 4 Division of Cancer and Stem Cells, Centre for Cancer Sciences, Biodiscovery Institute, School of Medicine, University of Nottingham, United Kingdom.; 5 Centre of Membrane Proteins and Receptors (COMPARE), University of Birmingham and University of Nottingham, Birmingham, United Kingdom.; 6 Center for Vascular Biology Research, Beth Israel Deaconess Medical Center, Harvard Medical School, Boston, Massachusetts, USA.; 7 Department of Biomedical Engineering, Boston University, Boston, Massachusetts, USA.; 8 Beijer Laboratory for Gene and Neuroscience Research, Department of Immunology, Genetics and Pathology, University of Uppsala, Uppsala, Sweden.; 9 Department of Surgery, University of Melbourne, Parkville, Victoria, Australia.; 10 Department of Ophthalmology, Royal Melbourne Hospital, Parkville, Victoria, Australia.

**Keywords:** Angiogenesis, Apoptosis

## Abstract

Aberrant, neovascular retinal blood vessel growth is a vision-threatening complication in ischemic retinal diseases. It is driven by retinal hypoxia frequently caused by capillary nonperfusion and endothelial cell (EC) loss. We investigated the role of EC apoptosis in this process using a mouse model of ischemic retinopathy, in which vessel closure and EC apoptosis cause capillary regression and retinal ischemia followed by neovascularization. Protecting ECs from apoptosis in this model did not prevent capillary closure or retinal ischemia. Nonetheless, it prevented the clearance of ECs from closed capillaries, delaying vessel regression and allowing ECs to persist in clusters throughout the ischemic zone. In response to hypoxia, these preserved ECs underwent a vessel sprouting response and rapidly reassembled into a functional vascular network. This alleviated retinal hypoxia, preventing subsequent pathogenic neovascularization. Vessel reassembly was not limited by VEGFA neutralization, suggesting it was not dependent on the excess VEGFA produced by the ischemic retina. Neutralization of ANG2 did not prevent vessel reassembly, but did impair subsequent angiogenic expansion of the reassembled vessels. Blockade of EC apoptosis may promote ischemic tissue revascularization by preserving ECs within ischemic tissue that retain the capacity to reassemble a functional network and rapidly restore blood supply.

## Graphical abstract

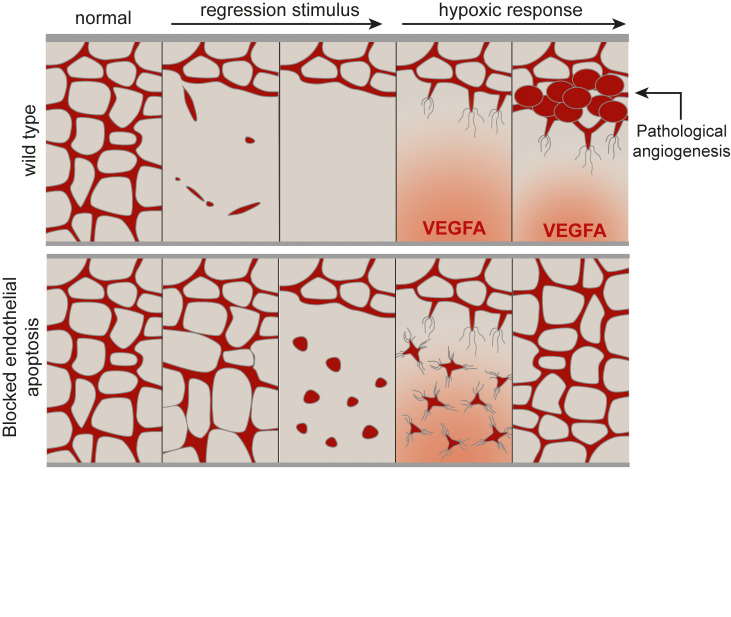

## Introduction

Angiogenesis is the growth of new blood vessels from preexisting vessels and occurs through a tightly regulated response of endothelial cells (ECs) to proangiogenic factors (1, 2). Insufficient vascular supply, which may arise due to impaired vessel growth (3), capillary loss (4), or vessel obstruction (5), causes tissue ischemia. While increased angiogenesis correlates with better outcomes in ischemic diseases like stroke (6), ischemia and the upregulation of VEGFA can drive aberrant angiogenesis, exacerbating certain diseases. This is particularly evident in retinal diseases, such as proliferative diabetic retinopathy, retinal vein occlusion, and retinopathy of prematurity, in which aberrant angiogenesis (neovascularization) increases the risk of severe vision loss and blindness (7). Current treatments for neovascular disease in the retina revolve around reducing the angiogenic stimulus either by decreasing the metabolic activity of the retina or by direct inhibition of VEGFA (8–12). While these approaches improve visual outcomes, many patients show either no response or a suboptimal response (8, 9). Capillary regression and the loss of ECs from the microvasculature is commonly associated with progression to neovascular disease in the retina and can occur in response to metabolic dysregulation (4), hyperoxia (13), or interrupted blood flow (14, 15). ECs die by apoptosis, a genetically programmed form of cell death (16), in settings where extensive vessel regression occurs (17, 18). Retinal capillaries in patients with diabetic retinopathy, for example, show elevated levels of EC apoptosis before progression to the proliferative phase (4, 19). Understanding the role of apoptosis in EC loss and vessel regression leading to retinal ischemia may therefore provide new insight into vascular changes associated with ischemia and potentially new avenues for preventing disease progression.

Apoptosis is regulated by 2 pathways, one consisting of BCL2 family proteins, the other consisting of cell surface “death receptors.” Previous studies have demonstrated a key role for the BCL2 family of proteins in the regulation of EC apoptosis (17). The BCL2 family consists of opposing factions of prosurvival and proapoptotic members (16), the balance of which determines whether the apoptosis effector proteins BAK and BAX become activated (20). Previous studies have shown that among the BCL2 family, the prosurvival protein MCL1 (21), the proapoptotic protein BIM (22, 23), and the 2 effector proteins BAK and BAX (24) have a central role in regulating EC apoptosis in the retina in vivo.

Vessel regression is a normal process necessary for establishing hierarchic vessel patterning during angiogenesis and the removal of redundant or damaged vascular networks (17, 18). During angiogenic vessel remodeling, local differences in blood flow shear between neighboring vessel segments determine which will be “pruned” away (25–27). This selective vessel pruning is driven by EC migration (25, 27, 28), does not cause ischemia, and does not require EC apoptosis (24). In contrast, apoptosis does appear responsible for capillary regression in other contexts, including those that leave tissues with insufficient vascular supply, causing ischemia. Exactly how apoptosis contributes to vessel loss in this context and the outcomes of preventing it are not well understood.

Here we have investigated the effect of blocking EC apoptosis in a mouse model of ischemia-induced neovascular disease (4), in which blocking apoptosis is known to prevent capillary loss and subsequent neovascularization (23). Our results show that EC apoptosis in this model is secondary to a vessel closure event that deprives downstream capillaries of blood flow. Rather than preventing this vessel closure or subsequent vessel degeneration, protecting ECs from apoptosis instead preserved ECs from these closed vessels within the ischemic region that were capable of rebuilding a functional vascular network in response to hypoxia-derived signals, restoring tissue oxygenation and mitigating the damaging effects of prolonged hypoxia on the retina.

## Results

### Blocking BCL2 pathway apoptosis results in EC survival and delayed vessel regression.

**Figure 1:**
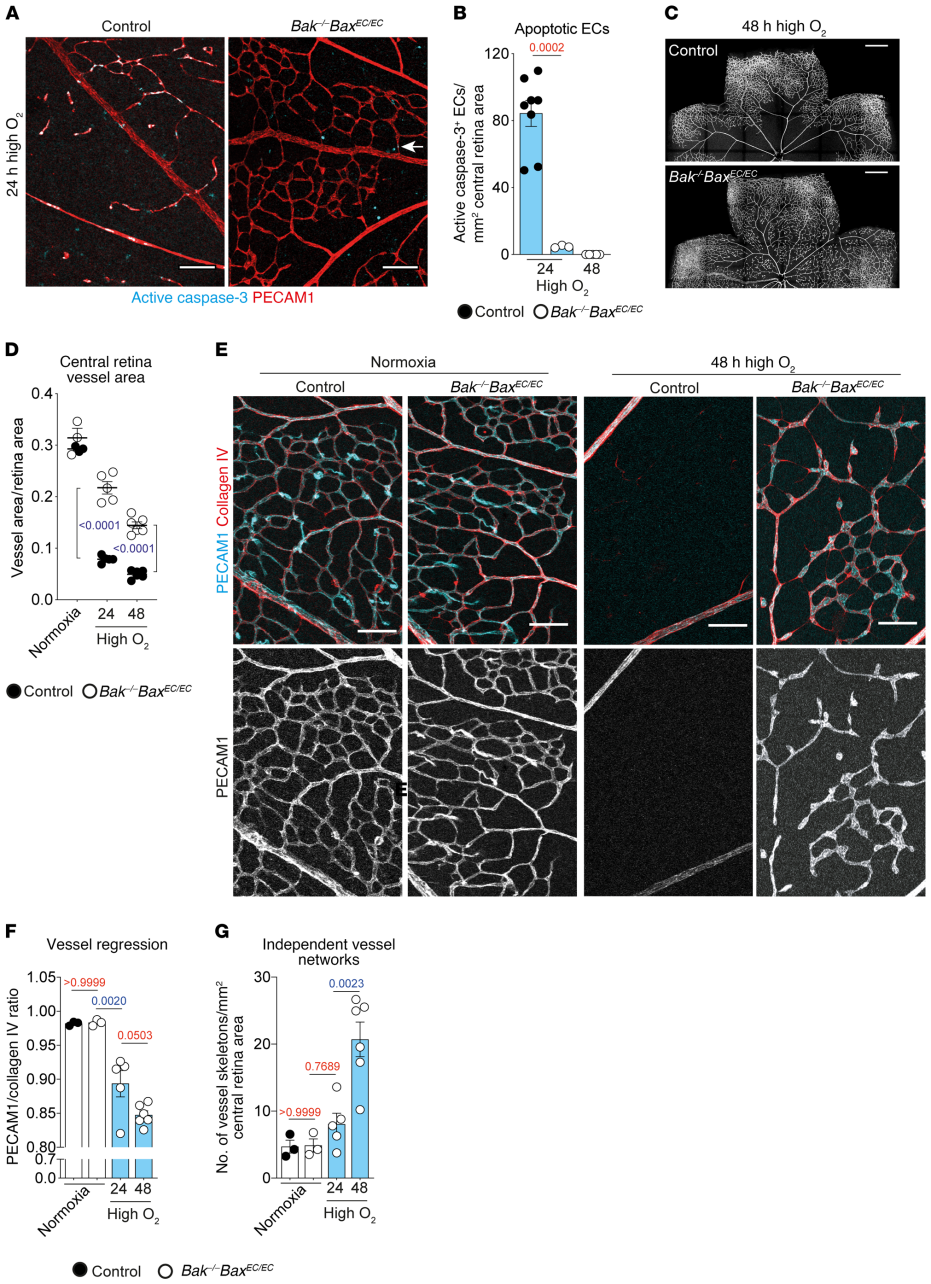
Blocking apoptosis prevents loss of ECs from retinas exposed to high oxygen. (**A** and **B**) Representative images and quantification of EC apoptosis visualized by active caspase-3 staining (cyan) and PECAM1 (red) in control (*n* = 8) and *Bak^–/–^ Bax^EC/EC^* (24 hours, *n* = 3; 48 hours, *n* = 5) retinas after 24 or 48 hours in high oxygen. Quantitative data from control mice exposed to high oxygen for 48 hours are not shown because there are no central retina capillaries remaining. Arrow indicates rare apoptotic EC in *Bak^–/–^ Bax^EC/EC^* retina. Scale bars: 100 μm. Student’s 2-tailed *t* test. (**C**) PECAM1 staining of control and *Bak^–/–^ Bax^EC/EC^* retinas after 48 hours in high oxygen. Scale bars: 500 μm. (**D**) Quantification of central retina vessel area in mice exposed to high oxygen for 24 hours (control, *n* = 4; *Bak^–/–^ Bax^EC/EC^*, *n* = 5) or 48 hours (control, *n* = 6; *Bak^–/–^ Bax^EC/EC^*, *n* = 6) compared with 8-day-old normoxic mice (control, *n* = 3; *Bak^–/–^ Bax^EC/EC^*, *n* = 3). Multiple *t* tests using Holm-Šidák correction for multiple comparisons. (**E**) PECAM1 (cyan) and collagen IV (red) staining within the central retina of control and *Bak^–/–^ Bax^EC/EC^* mice raised in room air (normoxia) or for 48 hours in high oxygen. Scale bars: 80 μm. (**F** and **G**) Quantification of vessel regression and network fragmentation in the central retina of *Bak^–/–^ Bax^EC/EC^* mice exposed to high oxygen for 24 hours (*n* = 5) or 48 hours (*n* = 6) compared with 8-day-old normoxic mice (control, *n* = 3; *Bak^–/–^ Bax^EC/EC^*, *n* = 3). Quantitative data from control mice exposed to high oxygen are not shown because there are no central retina capillaries remaining. One-way ANOVA with Tukey’s multiple-comparisons test. All data are mean ± SEM. Each circle represents 1 animal.

To determine whether EC apoptosis was responsible for vessel regression causing ischemia, we investigated its role in the oxygen-induced retinopathy (OIR) model (ref. 29 and Supplemental Figure 1A; supplemental material available online with this article; https://doi.org/10.1172/JCI127668DS1). In this model, transient exposure of mice to high oxygen causes the apoptotic death of ECs and consequent regression of retinal capillaries in the center of the retina, resulting in relative retinal hypoxia once the mice are returned to room air oxygen levels. This is followed by the development of abnormal vascular lesions that resemble those found in neovascular retinal diseases (4) and was developed as a model of retinopathy of prematurity. To block apoptosis, we used mice that we previously generated, in which apoptosis was inactivated in ECs through combined deletion of BAK and BAX (*Bak^–/–^ Bax^EC/EC^* mice [ref. 24]). *Bak^–/–^ Bax^EC/EC^* mice lack BAK in all tissues and BAX only in ECs, a necessary strategy because *Bak^–/–^* mice are viable, whereas most *Bak^^–/–^^ Bax^–/–^* double-null mice die at birth due to a range of birth defects (30). Relevant to the age of mice studied here, *Bak^–/–^ Bax^EC/EC^* mice develop a normal retinal vascular network in the first few weeks after birth (24). After 24 hours of exposure to high oxygen, control-genotype mice contained extensive numbers of apoptotic ECs (active caspase-3^+^ PECAM1^+^ cells). In contrast, retinas from *Bak^–/–^ Bax^EC/EC^* mice were almost completely devoid of apoptotic ECs after 24 or 48 hours of oxygen exposure (Figure 1, A and B). By contrast, other forms of programmed cell death, namely death receptor–mediated apoptosis (caspase-8–dependent) and MLKL-dependent necroptosis, were dispensable for vaso-obliteration, as *caspase-8^–/–^ Mlkl^–/–^* double-knockout mice underwent normal vaso-obliteration when exposed to high oxygen (Supplemental Figure 1, B and C). These results confirm the central role of the BCL2-regulated apoptosis pathway in the apoptotic response of ECs in the OIR model.

As a result of blocking apoptosis, *Bak^–/–^ Bax^EC/EC^* retinas contained significantly more vasculature than retinas of control littermates following 24–48 hours of high oxygen exposure (Figure 1C and Supplemental Figure 1D). Despite this, the vessel area in *Bak^–/–^ Bax^EC/EC^* retinas declined with increasing duration of high oxygen exposure (Figure 1D). This loss of vasculature was due to vessel regression based on the reduced occupancy of collagen IV^+^ vascular basement membranes with PECAM1^+^ ECs (ref. 31; Figure 1, E and F; and Supplemental Figure 1E). By 48 hours of exposure to high oxygen, ongoing vessel regression had caused the network in *Bak^–/–^ Bax^EC/EC^* retinas to degenerate into isolated vessel fragments and endothelial clusters that in many cases remained linked by empty collagen IV sleeves (Figure 1, E and G), evidence that they were once part of an interconnected vascular network.

The *Tie2-Cre* transgene used to generate *Bak^–/–^ Bax^EC/EC^* mice is active in hematopoietic cells as well as ECs. The *Cdh5-CreERT2* transgene (32) is active in ECs but not hematopoietic cells following postnatal administration of tamoxifen (21). Postnatal deletion of *Bax* only from ECs using this Cre (*Bak^–/–^ Bax^iEC/iEC^* mice) recapitulated the fragmented vascular phenotype after 48 hours of high oxygen exposure (Supplemental Figure 1, F–H). This result confirmed that the fragmented vascular phenotype is due to apoptosis blockade specifically in ECs. Therefore, blocking apoptosis in ECs does not prevent vessel regression triggered by exposure to high oxygen.

### EC apoptosis–independent loss of blood flow precedes vessel regression.

We sought to determine why apoptosis suppression could not prevent the loss of retinal capillary network integrity following high oxygen exposure by investigating the causes of EC apoptosis and vessel regression. VEGFA promotes EC survival via the BCL2 pathway (33, 34). It is downregulated during exposure to high oxygen, and this has been suggested to contribute to vessel regression in the OIR model (35–37). VEGFR2 is the major VEGFA receptor responsible for survival signaling by VEGFA (38). Using tamoxifen-inducible, EC-specific *Vegfr2* mutants described previously (39), we found that reducing VEGFR2 through tamoxifen administration from postnatal day 7 (P7; the age at which retinal vessels are susceptible to high oxygen) did not result in widespread loss of central retina capillaries when analyzed at P10 (Supplemental Figure 2, A and B). This result suggests that a reduction in VEGFA activity alone is not sufficient to replicate the vaso-obliteration that occurs in the OIR model and is consistent with previous findings (40, 41).

**Figure 2:**
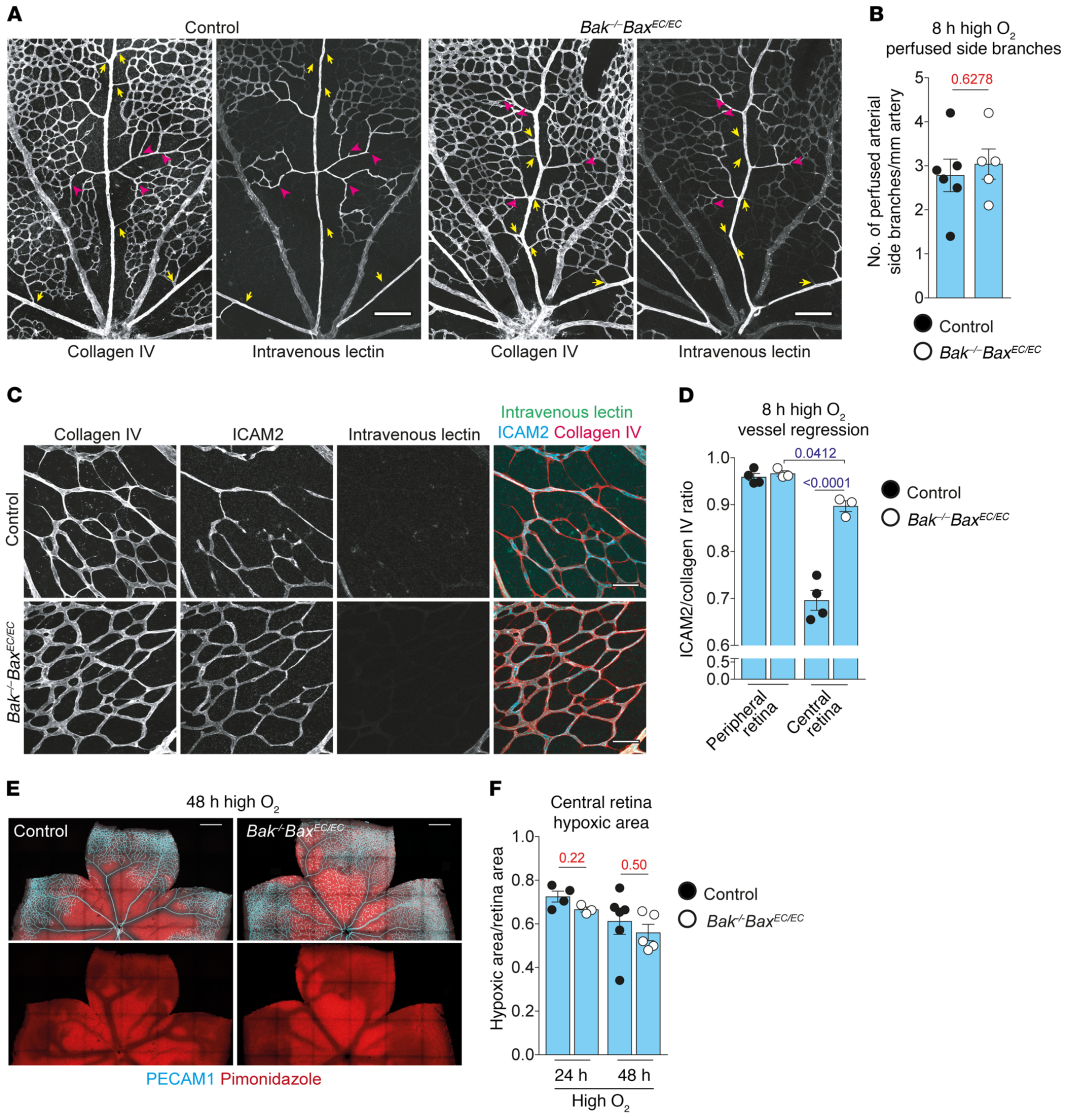
Blocking apoptosis delays, but does not prevent, vessel regression. (**A**) Collagen IV and perfused lectin staining in control and *Bak^–/–^ Bax^EC/EC^* central retinas. Yellow arrows indicate arterial side-branch closure; pink arrowheads indicate representative downstream vessel closure points. Scale bars: 100 μm. (**B**) Perfused arterial side branches in control (*n* = 6) and *Bak^–/–^ Bax^EC/EC^* mice (*n* = 5). Student’s 2-tailed *t* test. (**C**) Representative regions of nonperfused capillaries from the central retinas of control and *Bak^–/–^ Bax^EC/EC^* mice stained for collagen IV (red), ICAM2 (cyan), and perfused lectin (green). Scale bars: 50 μm. (**D**) Vessel regression in the peripheral and central retina capillaries from control (*n* = 4) and *Bak^–/–^ Bax^EC/EC^* (*n* = 3) mice. Two-way ANOVA using Tukey’s multiple-comparisons test. (**E**) Hypoxia visualized by pimonidazole (red) staining and ECs by PECAM1 (cyan) in control and *Bak^–/–^ Bax^EC/EC^* retinas following 48 hours of high oxygen exposure. Scale bars: 500 μm. (**F**) Central retina hypoxic area in mice exposed to high oxygen for 24 hours (control, *n* = 4; *Bak^–/–^ Bax^EC/EC^*, *n* = 3) or 48 hours (control, *n* = 6; *Bak^–/–^ Bax^EC/EC^*, *n* = 5). Multiple *t* tests using Holm-Šidák correction for multiple comparisons. Animals in **A**–**D** were exposed to high oxygen for 8 hours. All data are mean ± SEM. Each circle represents 1 animal.

Reduced blood flow triggers the regression of immature retinal vessels (28, 41), and EC apoptosis in OIR has previously been proposed to occur secondary to loss of blood flow (41, 42). Widespread EC apoptosis was evident in control mice but not *Bak^–/–^ Bax^EC/EC^* mice 8 hours after onset of exposure to high oxygen (Supplemental Figure 2, C and D). With few exceptions, apoptosis was localized to nonperfused vessels based on costaining for active caspase-3 and intravenously perfused *Lycopersicon esculentum* lectin (Supplemental Figure 2E), which binds to and marks ECs in patent vessels. Loss of flow was mostly observed downstream of arterial side branches that had closed (Figure 2A). This pattern of vessel closure was observed in both control and *Bak^–/–^ Bax^EC/EC^* mice (Figure 2A). The number of perfused arterial side branches remaining at 8 hours was not different between *Bak^–/–^ Bax^EC/EC^* mice and controls (Figure 2, A and B). This demonstrates that apoptosis is not responsible for hyperoxia-induced arterial side-branch closure or deprivation of downstream capillaries of blood flow. Nonetheless, the regression of closed arterial side branches was delayed in the absence of apoptosis, as fewer nonperfused side branches in the *Bak^–/–^ Bax^EC/EC^* mice had fully disconnected their lumens from the artery by 8 hours (Supplemental Figure 2, F and G). There was also less regression occurring in nonperfused *Bak^–/–^ Bax^EC/EC^* capillaries relative to controls (Figure 2, C and D), although the nonperfused capillaries in mutants still showed slightly elevated levels of regression relative to the peripheral capillary plexus region that is unaffected by high oxygen exposure (Figure 2D).

Retinas vaso-obliterated by exposure to high oxygen become hypoxic on return to room air owing to the loss of the central capillary network (43), and this was observed in control mice following either 24 or 48 hours of exposure to high oxygen (Figure 2, E and F). The extent of hypoxia in littermate *Bak^–/–^ Bax^EC/EC^* retinas was equivalent to that in the controls, consistent with loss of blood flow to and fragmentation of the central retinal capillaries (Figure 2, E and F). These data suggest that vessel regression still occurs in the absence of apoptosis, likely as a result of blood flow loss.

### Preserved Bak^–/–^ Bax^EC/EC^ ECs rapidly reassemble the vessel network in response to hypoxia.

**Figure 3:**
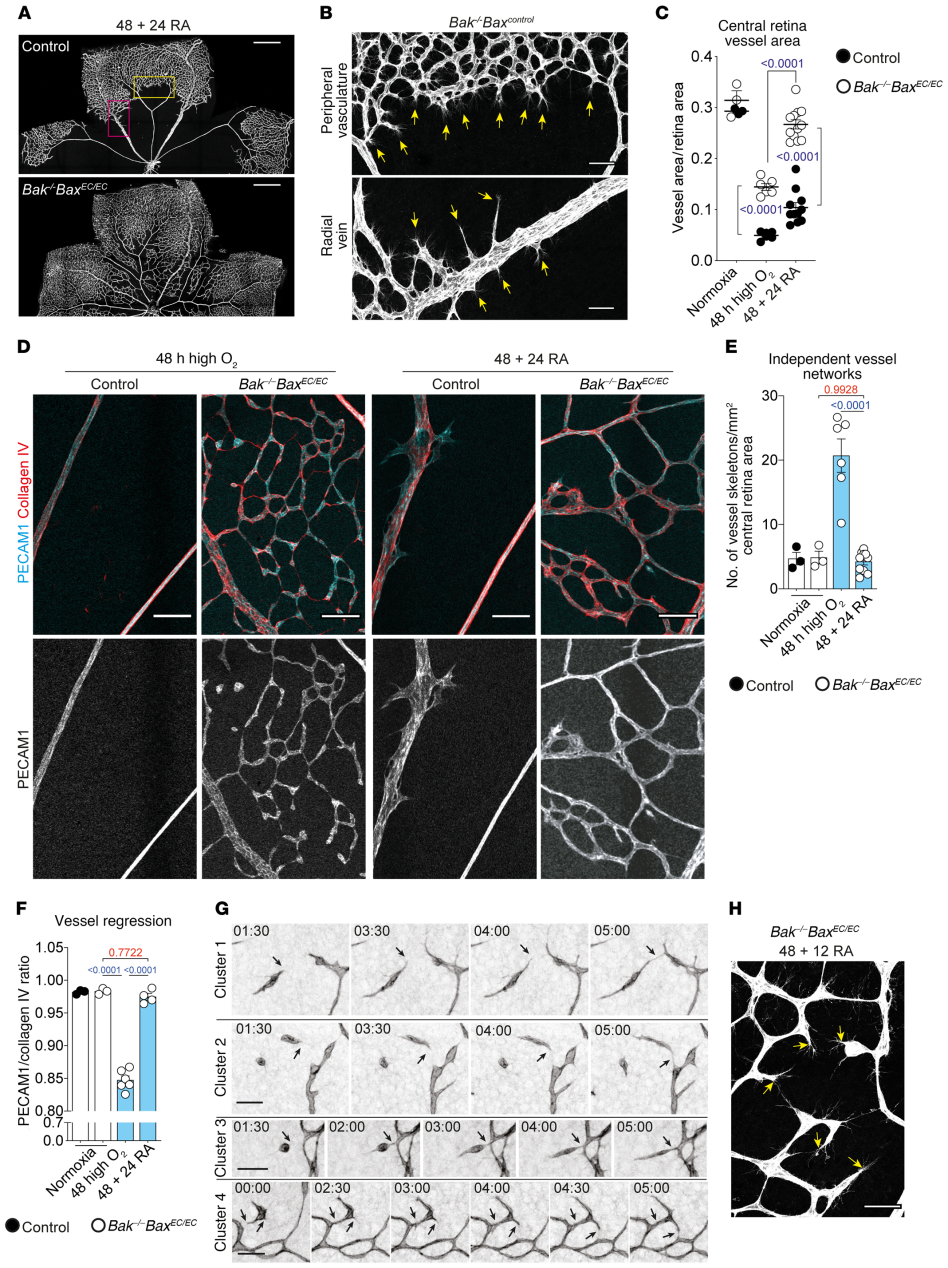
Isolated ECs protected from apoptosis rapidly reassemble to revascularize hypoxic retinas. (**A** and **B**) PECAM1 staining of control and *Bak^–/–^ Bax^EC/EC^* retinas exposed to high oxygen for 48 hours followed by 24 hours in room air (48 + 24 RA). Scale bars: 500 μm (**A**); 60 μm (**B**). Arrows indicate sprouting vessels. Boxed areas are enlarged in **B** (top, yellow box; bottom, pink box). (**C**) Vessel area in 48 + 24 RA control (*n* = 11) and *Bak^–/–^ Bax^EC/EC^* (*n* = 12) central retinas. Data for normoxic mice or mice exposed to high oxygen for 48 hours from Figure 1D are included for comparison. Two-way ANOVA using Tukey’s multiple-comparisons test. (**D**) Central retinal vasculature from control and *Bak^–/–^ Bax^EC/EC^* mice exposed to 48 hours of high oxygen or 48 + 24 RA stained for collagen IV (red) and PECAM1 (cyan). Scale bars: 80 μm. (**E** and **F**) Network fragmentation (*n* = 10) and vessel regression (*n* = 4) in 48 + 24 RA *Bak^–/–^ Bax^EC/EC^* central retinas. Data for normoxic mice or mice exposed to 48 hours of high oxygen from Figure 1, F and G, are included for comparison. One-way ANOVA with Tukey’s multiple-comparisons test. Quantitative data from control mice exposed to high oxygen are not shown because there are no central retina capillaries remaining. (**G**) Static images from live-imaging retinal explants showing vessels reassembling starting 12 hours after return to room air following 48 hours of exposure to high oxygen. Four independent clusters are shown. Time stamp is hh:mm (time 0 = 12 hours after return to room air). Arrows indicate where sprouts form new connections. Scale bars: 50 μm. (**H**) Sprouting clusters from a 48 + 12 RA *Bak^–/–^ Bax^EC/EC^* retina. Scale bar: 50 μm. Arrows indicate filopodial projections. All data are mean ± SEM. Each circle represents 1 animal.

In WT mice, the onset of hypoxia following return to room air induces a sprouting angiogenic response from the remaining vasculature that gradually revascularizes the central retina through centripetal growth of the network (44, 45). To determine whether the preserved ECs in the central retina of *Bak^–/–^ Bax^EC/EC^* mice could also respond to hypoxia, mice were exposed to high oxygen for 48 hours to fragment the vascular network, returned to room air to induce hypoxia in the avascular retina, then examined 24 hours later (referred to as 48 + 24 RA) (Supplemental Figure 3A). As expected, new-vessel growth from the peripheral plexus and radial veins in control mice only partially revascularized the retina 24 hours after onset of hypoxia (Figure 3, A–C). In contrast, *Bak^–/–^ Bax^EC/EC^* retinas showed a significant increase in vessel area accompanied by the cessation of vessel regression and reestablishment of an interconnected vascular network (Figure 3, A and C–F).

To understand how an intact vascular network was reestablished in the *Bak^–/–^ Bax^EC/EC^* mice, we performed time-lapse imaging to track the fate of the preserved ECs. For this, the Cre-inducible, cell membrane–targeted EGFP reporter allele *mTmG* (46) was intercrossed with the tamoxifen-inducible *Bak^–/–^ Bax^iEC/iEC^* mice. Following tamoxifen administration, *Bak^–/–^ Bax^iEC/iEC^ mTmG^ki/+^* pups were exposed to high oxygen for 48 hours, returned to room air for 12 hours to initiate the hypoxic response, and then retinas were explanted and immediately time-lapse-imaged at 30-minute intervals for 5 hours (Supplemental Figure 3, B and C). Time-lapse imaging showed that ECs in the isolated clusters adopted a migratory phenotype consistent with tip cell activity normally seen during sprouting angiogenesis (1). Through this, the isolated ECs actively reestablished connections with their neighbors, reassembling the network (Figure 3G and Supplemental Videos 1–3, black arrows). Vessel sprouts establishing new connections were also observed from already intact vessels (Supplemental Videos 1 and 2, red arrows). In many cases, migrating ECs extended multiple filopodial projections suggesting de novo pathfinding similar to the sprouting angiogenesis seen in control mice (Figure 3H, Supplemental Video 3, red arrows). In other cases, ECs appeared to track along predetermined paths (Supplemental Video 3, blue arrows). These data show that isolated EC clusters protected from apoptosis are active participants in the reestablishment of an intact vascular network during revascularization of the ischemic retina.

**Figure 4:**
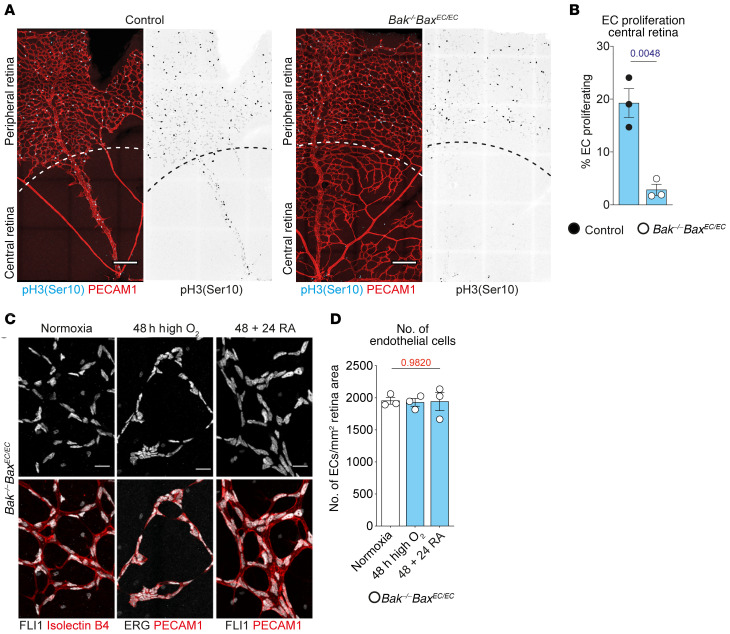
Vessel reassembly in *Bak^–/–^ Bax^EC/EC^* retinas uses preexisting, apoptosis-protected ECs. (**A**) EC proliferation in 48 + 24 RA control and *Bak^–/–^ Bax^EC/EC^* retinas visualized by staining for phospho–histone H3 (Ser10) (cyan) and PECAM1 (red). Dashed lines demarcate boundary between peripheral and central retina. Scale bars: 200 μm. (**B**) Quantification of proliferating ECs within central retina of 48 + 24 RA control (*n* = 3) and *Bak^–/–^ Bax^EC/EC^* (*n* = 3) mice. Student’s 2-tailed *t* test. (**C** and **D**) Representative images and quantification of EC number in *Bak^–/–^ Bax^EC/EC^* central retina vessels from normoxic mice (*n* = 3) or mice exposed to 48 hours of high oxygen alone (*n* = 3) or with 24 hours of recovery in room air (48 + 24 RA) (*n* = 3). EC number is quantified based on EC nuclei (costaining of FLI1 or ERG with EC marker PECAM1 or isolectin B4). Quantitative data from control mice are not shown because there are no central retina capillaries remaining following exposure to high oxygen. Scale bars: 20 μm. One-way ANOVA. All data are mean ± SEM. Each circle represents 1 animal.

During normal sprouting angiogenesis in the retina, new-vessel growth is coupled to EC proliferation (47). Control and *Bak^–/–^ Bax^EC/EC^* retinas were stained for phospho–histone H3 (Ser10) to identify proliferating ECs 24 hours after return to room air. In control retinas, EC proliferation accompanied regrowth of the central retinal vasculature (Figure 4, A and B, and Supplemental Figure 3D). In contrast, reassembling vessels in the center of *Bak^–/–^ Bax^EC/EC^* retinas contained few proliferating ECs (Figure 4, A and B). EC proliferation in the peripheral vasculature was similar between control and *Bak^–/–^ Bax^EC/EC^* mice, indicating that there was not a general EC proliferation defect in the mutants (Supplemental Figure 3E).

To determine whether EC number changed during the process of regression and reassembly, we quantified EC number in normal (normoxic), fragmented (48 hours high oxygen), and reassembled (48 + 24 RA) *Bak^–/–^ Bax^EC/EC^* central retina vessels, using EC nuclei markers FLI1 and ERG. FLI1 was downregulated in ECs of fragmented vessels (Supplemental Figure 3F); therefore ERG was used to quantify EC number under this condition. We found that the number of ECs in the central retina of *Bak^–/–^ Bax^EC/EC^* mice remained constant between normoxic, fragmented, and reassembled vessels (Figure 4, C and D). Collectively, these data show that blocking apoptosis prevents the loss of ECs from the retina and that reassembly of these cells back into interconnected vessels does not require the production of new ECs.

### The extent of network fragmentation influences vessel reassembly.

The degree of network fragmentation in *Bak^–/–^ Bax^EC/EC^* mice was proportional to the time spent in the high-oxygen environment. Those mice exposed to high oxygen for 24 hours showed less extensive vessel regression and network fragmentation than those exposed for 48 hours (Figure 1, F and G, and Supplemental Figure 4A). When mice exposed to high oxygen for 24 hours were returned to room air for a further 24 hours to stimulate hypoxia-driven vessel reassembly (24 + 24 RA), the vascular area, vessel width, and network branch points were all closer to those of mice raised in normoxia than was observed in the *Bak^–/–^ Bax^EC/EC^* mice exposed to 48 + 24 RA (Supplemental Figure 4, B–D). This finding suggests that the sooner tissue hypoxia manifests after flow interruption, the more efficiently apoptosis-resistant ECs can reassemble a functional vascular network.

### Vessel reassembly facilitated by the blocking of apoptosis reverses retinal hypoxia, and associated pathological responses.

**Figure 5:**
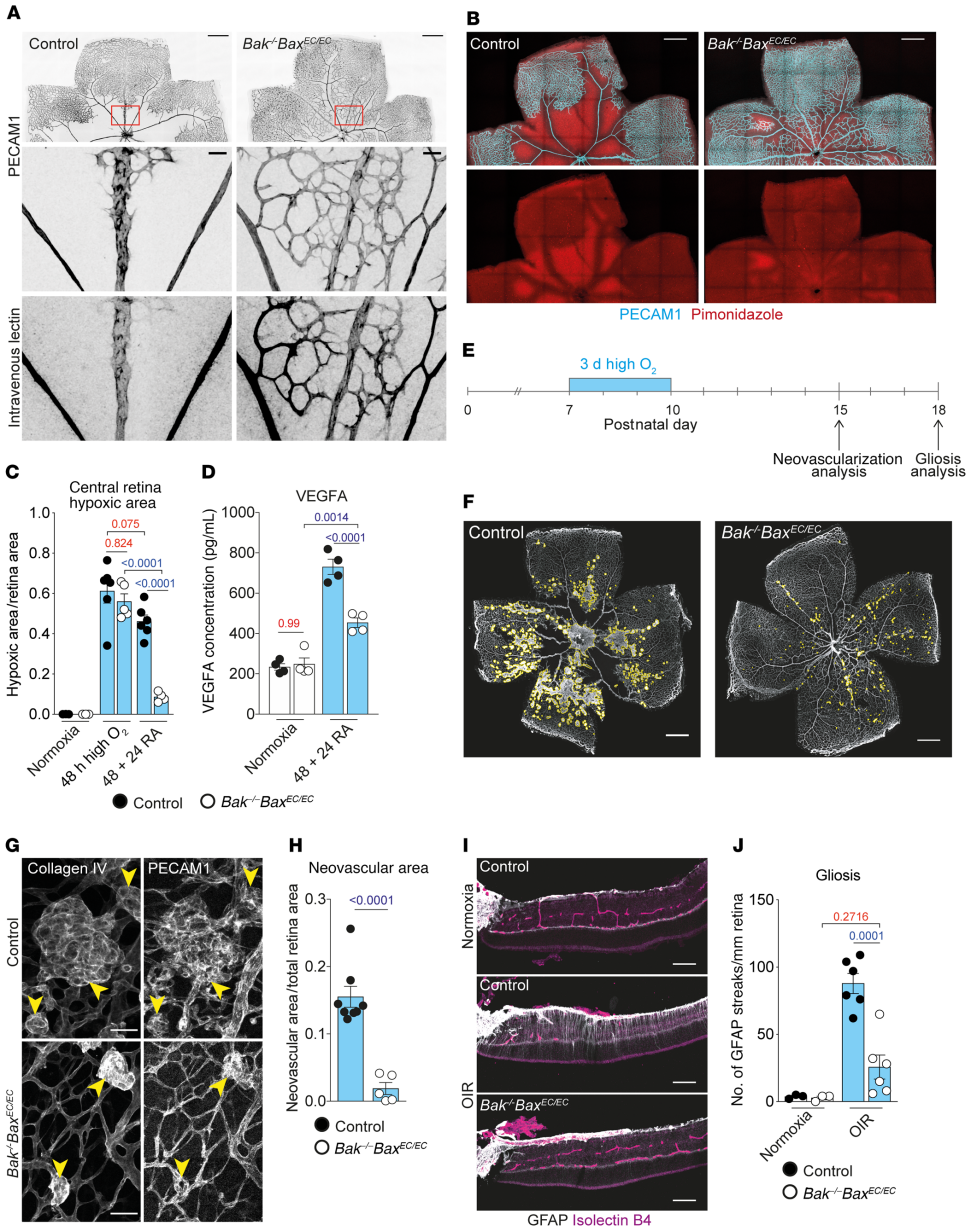
Reassembled vessels in *Bak^–/–^ Bax^EC/EC^* retinas are functional and limit neovascularization and retinal injury. (**A**) 48 + 24 RA control and *Bak^–/–^ Bax^EC/EC^* retinas perfused i.v. with lectin and stained for PECAM1. (**B**) Hypoxia visualized by pimonidazole (red) staining (costained with PECAM1, cyan) in control and *Bak^–/–^ Bax^EC/EC^* retinas exposed to high oxygen for 48 hours followed by 24 hours in room air (48 + 24 RA). Scale bars: 500 μm. (**C**) Central retina hypoxia in P8 normoxic mice (control, *n* = 3; *Bak^–/–^ Bax^EC/EC^*, *n* = 3) following 48 hours in high oxygen (control, *n* = 6; *Bak^–/–^ Bax^EC/EC^*, *n* = 5) or 48 + 24 RA (control, *n* = 6; *Bak^–/–^ Bax^EC/EC^*, *n* = 4). Two-way ANOVA using Tukey’s multiple-comparisons test. (**D**) Quantification of VEGFA protein in whole retina extracts from 48 + 24 RA control (*n* = 4) and *Bak^–/–^ Bax^EC/EC^* (*n* = 4) mice and age-matched normoxic controls (control, *n* = 4; *Bak^–/–^ Bax^EC/EC^*, *n* = 4). Two-way ANOVA using Tukey’s multiple-comparisons test. (**E**) Experimental overview of OIR procedure used in **F**–**J**. (**F**–**H**) Representative examples and quantification of neovascular area in P15 control (*n* = 8) and *Bak^–/–^ Bax^EC/EC^* (*n* = 5) retinas stained for collagen IV and PECAM1. Yellow lines outline neovascular lesions (**F**); arrowheads indicate glomerular-like lesions (**G**). Scale bars: 500 μm (**F**); 50 μm (**G**). Student’s 2-tailed *t* test. (**I** and **J**) Representative images and quantification of Müller cell gliosis visualized by GFAP (gray) staining comparing mice subjected to OIR (control, *n* = 6; *Bak^–/–^ Bax^EC/EC^*, *n* = 6) and age-matched controls raised in room air (normoxia; control, *n* = 2; *Bak^–/–^ Bax^EC/EC^*, *n* = 2). Isolectin B4 labels ECs (magenta). Scale bars: 100 μm. Two-way ANOVA with Tukey’s multiple-comparisons test. All data are mean ± SEM. Each circle represents 1 animal.

To assess whether reassembled vessels were functional, we investigated vessel perfusion and retinal hypoxia in *Bak^–/–^ Bax^EC/EC^* and control mice subjected to 48 + 24 RA. Lectin perfusion showed that most reassembled vessels in *Bak^–/–^ Bax^EC/EC^* retinas were patent and perfused 24 hours after return to room air (Figure 5A). Consistent with this, there was significantly less hypoxia in the central retinas of *Bak^–/–^ Bax^EC/EC^* mice compared with similarly treated controls (Figure 5, B and C). These mice also displayed less retinal hypoxia than *Bak^–/–^ Bax^EC/EC^* retinas still in the fragmented state immediately following exposure to 48 hours of high oxygen alone (Figure 5, B and C). Retinal hypoxia in the OIR model following return to room air results in increased expression of VEGFA (36, 48). Accordingly, VEGFA protein levels increased substantially in control mice 24 hours after return to room air, but this was significantly reduced in *Bak^–/–^ Bax^EC/EC^* retinas (Figure 5D). This was consistent with there being less hypoxia as a result of reassembly of the vascular network and restoration of the vascular supply. As neovascular lesion formation is dependent on VEGFA (44, 45, 49), we assessed whether the reduction in hypoxia-induced VEGFA brought about by vessel reassembly would also translate to a reduction in neovascular lesion formation. Neovascular lesions have a glomerular vessel structure, distinct from the normal branched vessel network pattern. These lesions stained brightly for the basement membrane protein collagen IV (Figure 5G). The strong contrast in collagen IV signals between normal and neovascular vessels enabled us to quantify neovascular lesions as bright, globular collagen IV structures. Whereas control mice exposed to high oxygen and returned to room air for 5 days developed extensive neovascular lesion area, this was significantly reduced in *Bak^–/–^ Bax^EC/EC^* retinas (Figure 5, E–H). Additionally, Müller cell gliosis, an indicator of retinal stress, was significantly reduced in *Bak^–/–^ Bax^EC/EC^* mice compared with the controls (Figure 5, I and J). These findings demonstrate that reassembled vessel networks are functional and prevent pathological consequences of prolonged tissue hypoxia in the retina.

### Reducing VEGFA levels does not prevent vessel reassembly or its suppression of neovascular lesion formation.

**Figure 6:**
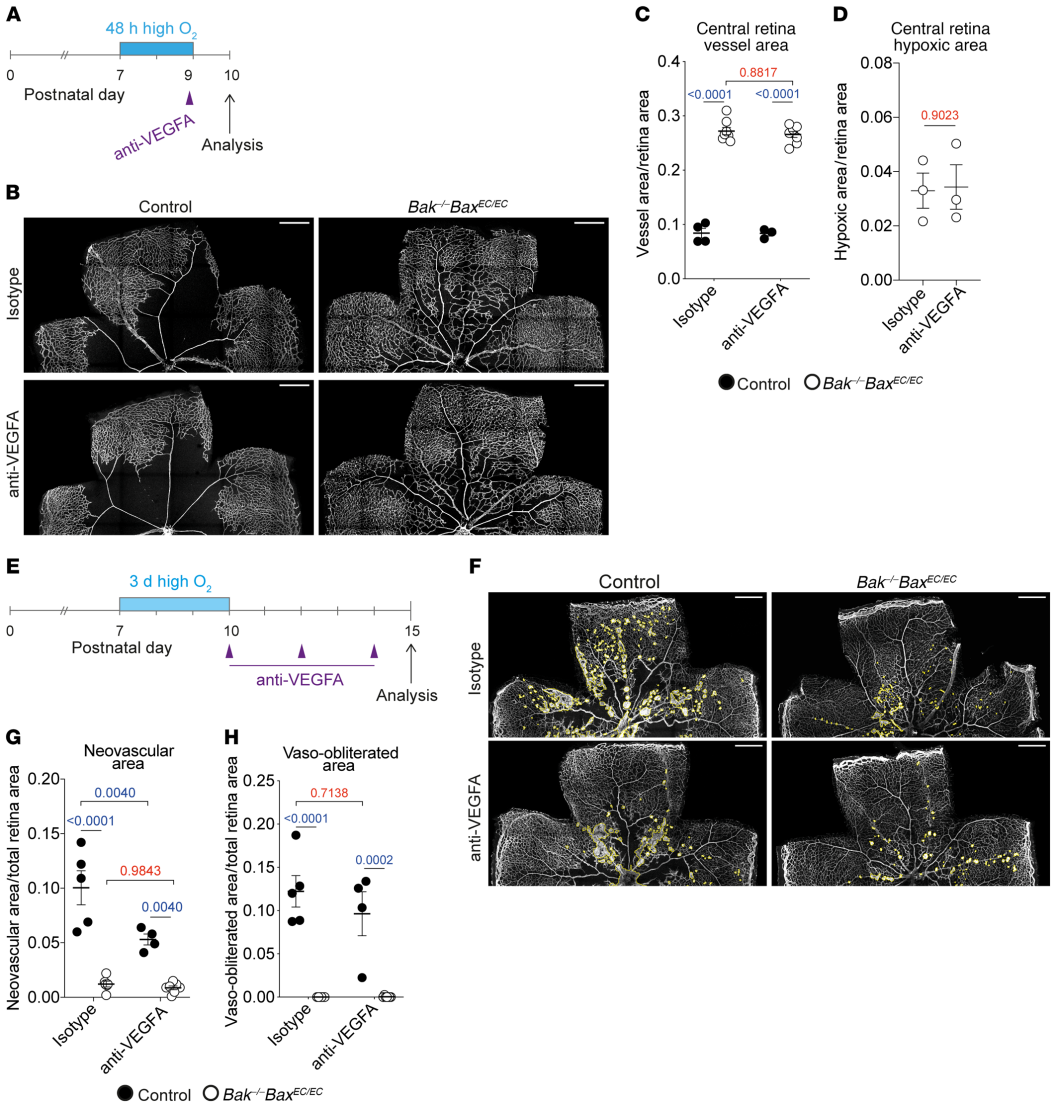
Vessel reassembly is insensitive to VEGFA neutralization. (**A**) Experimental overview of mice analyzed in **B**–**D**. (**B** and **C**) Representative images and quantification of central retinal vasculature in mice subjected to the time course shown in **A** and treated with isotype control (control, *n* = 4; *Bak^–/–^ Bax^EC/EC^*, *n* = 8) or anti-VEGFA (control, *n* = 3; *Bak^–/–^ Bax^EC/EC^*, *n* = 8). Stained for PECAM1. Scale bars: 500 μm. Two-way ANOVA with Tukey’s multiple-comparisons test. (**D**) Quantification of central retina hypoxic area in *Bak^–/–^ Bax^EC/EC^* mice subjected to the time course shown in **A** and treated with isotype control (*n* = 3) or anti-VEGFA (*n* = 3). Student’s 2-tailed *t* test. (**E**) Experimental overview of mice analyzed in **F**–**H**. (**F**) Representative examples of neovascularization (yellow outline) in control and *Bak^–/–^ Bax^EC/EC^* retinas treated with anti-VEGFA or isotype control antibodies. Scale bars: 500 μm. (**G**) Quantification of neovascular area in retinas from control (isotype, *n* = 5; anti-VEGFA, *n* = 4) and *Bak^–/–^ Bax^EC/EC^* mice (isotype, *n* = 6; anti-VEGFA, *n* = 7). Two-way ANOVA with Tukey’s multiple-comparisons test. (**H**) Quantification of vaso-obliterated area in retinas from control (isotype, *n* = 5; anti-VEGFA, *n* = 4) and *Bak^–/–^ Bax^EC/EC^* mice (isotype, *n* = 6; anti-VEGFA, *n* = 7). Two-way ANOVA with Tukey’s multiple-comparisons test. All data are mean ± SEM. Each circle represents 1 animal.

As elevated VEGFA drives aberrant angiogenesis in ischemic retinas, we investigated whether it was also necessary for the vessel reassembly that occurred in the absence of EC apoptosis. To test this, *Bak^–/–^ Bax^EC/EC^* mice were exposed to 48 + 24 RA to induce network fragmentation and reassembly and were treated with either a VEGFA-neutralizing antibody (50) or isotype control on return to room air (Figure 6A). VEGFA neutralization did not appear to prevent vessel reassembly, as no difference was observed in central retinal vascular area between mice treated with anti-VEGFA and those treated with isotype control antibody (Figure 6, B and C). Supporting this, the hypoxic area in the central retina was not different in *Bak^–/–^ Bax^EC/EC^* mice treated with anti-VEGFA versus isotype control antibody (Figure 6D). We confirmed that the systemically delivered antibodies were neutralizing VEGFA in the retinal vessels by staining for ESM1 (Supplemental Figure 5, A–C). The expression of ESM1 is dependent on VEGFA (51). Vessel reassembly proceeded in anti-VEGFA–treated mice even under conditions of high VEGFA signaling inhibition, in which ESM1 expression was reduced by 94% (Supplemental Figure 5, A–E). VEGFA inhibition under conditions that reduced neovascular lesion area by 50% in control genotype mice did not interfere with the ability of vessel reassembly to prevent neovascular lesion formation 5 days after return to room air (Figure 6, E–G). In contrast, vessel reassembly in *Bak^–/–^ Bax^EC/EC^* mice was effective at reducing neovascular area by 88% (Figure 6G).

The mature retinal vasculature consists of 3 layers: superficial, middle, and deep. *Bak^–/–^ Bax^EC/EC^* mice had established more extensive vessel networks in these layers than control mice 5 days after return to room air, evident from the extent of vaso-obliterated area repair (Figure 6, F and H) and the vessel area in these layers (Supplemental Figure 5, F–H). None of this was affected when VEGFA was neutralized in mice of either genotype (Figure 6H and Supplemental Figure 5, F–H). Taken together, these data show that vessel reassembly in the absence of EC apoptosis is not dependent on abnormally high VEGFA levels or impeded when VEGFA levels are reduced to a level sufficient to suppress aberrant neovascularization or ESM1 expression.

### Reducing ANG2 does not prevent vessel reassembly, but does impair angiogenic expansion of reassembled vessels.

**Figure 7:**
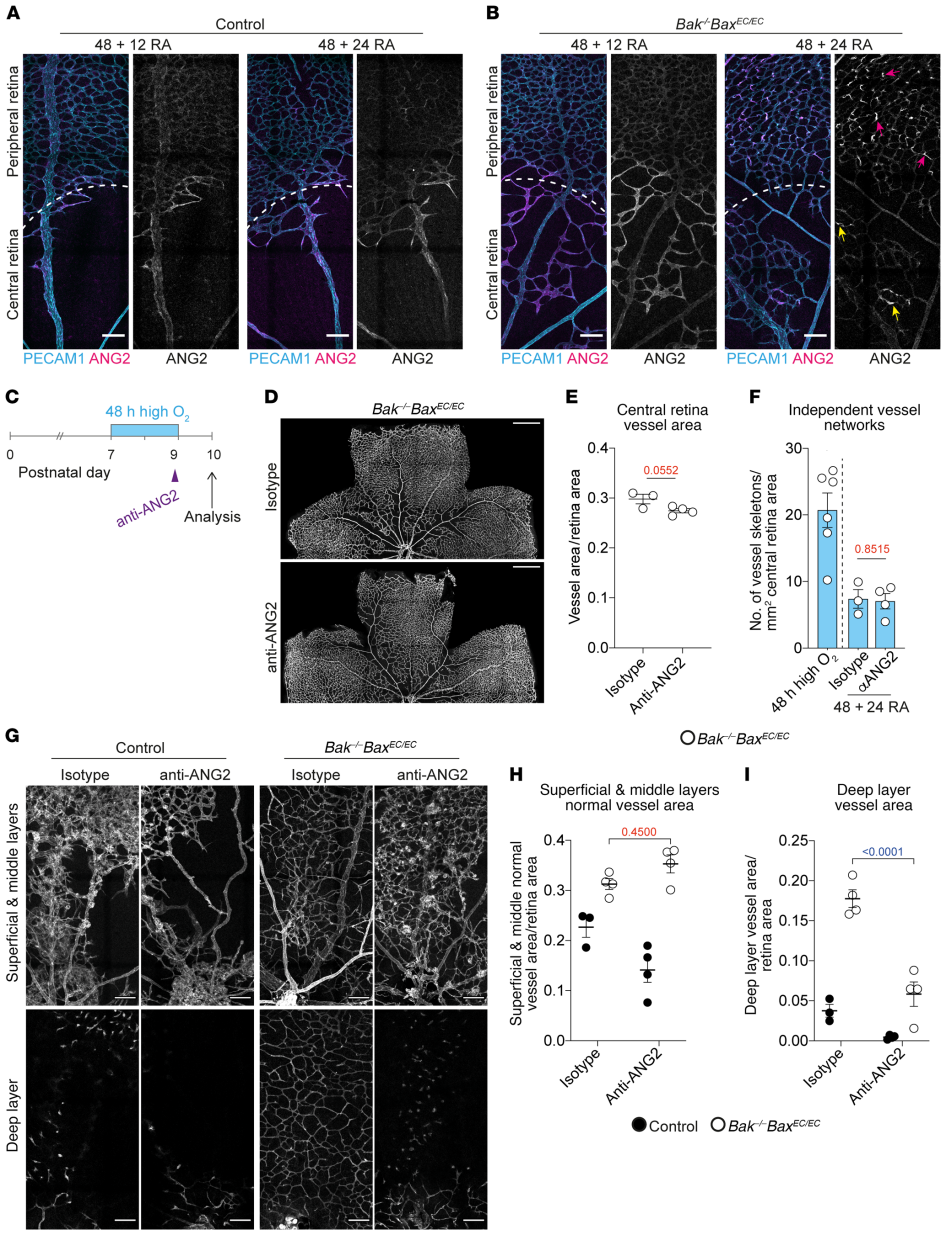
ANG2 is not required for vessel reassembly but is required for expansion of reassembled network. (**A** and **B**) Representative images of ANG2 (magenta, gray) expression in control and *Bak^–/–^ Bax^EC/EC^* retinas exposed to high oxygen for 48 hours followed by return to room air for 12 hours (+ 12 RA) or 24 hours (+ 24 RA). Costained with PECAM1 (cyan). Pink arrows indicate ANG2^+^ downward sprouts; yellow arrows indicate patches of ANG2^+^ vessels. Scale bars: 100 μm. (**C**) Experimental overview of mice analyzed in **D**–**F**. (**D** and **E**) Representative images and quantification of central retinal vasculature in *Bak^–/–^ Bax^EC/EC^* mice subjected to the time course shown in **C** and treated with isotype control (*n* = 3) or anti-ANG2 (*n* = 4). Stained for PECAM1. Scale bars: 500 μm. Student’s 2-tailed *t* test. (**F**) Quantification of network fragmentation in the central retina of *Bak^–/–^ Bax^EC/EC^* mice subjected to the time course shown in **C** and treated with isotype control (*n* = 3) or anti-ANG2 (*n* = 4). Data for *Bak^–/–^ Bax^EC/EC^* mice exposed to 48 hours of high oxygen from Figure 1G are shown for comparison. Student’s 2-tailed *t* test. (**G**–**I**) Representative images and quantification of vascular area in separate layers from the same field of view of the central retinas of control (isotype control, *n* = 3; anti-ANG2, *n* = 4) and *Bak^–/–^ Bax^EC/EC^* mice (isotype control, *n* = 4; anti-ANG2, *n* = 4). Scale bars: 100 μm. Two-way ANOVA with Tukey’s multiple-comparisons test. All data are mean ± SEM. Each circle represents 1 animal.

Our data show that vessel reassembly occurs in response to hypoxia by cells that exhibit hallmark features of endothelial tip cells. ANG2 is highly expressed in tip cells (52–54) and is essential for sprouting angiogenesis in the retina (53, 55, 56). ANG2 is upregulated in ECs by hypoxia (57–60), including in the OIR model (57). Given this, we sought to determine whether ANG2 was necessary for vessel reassembly. To investigate ANG2 expression in reassembling vessels, control and *Bak^–/–^ Bax^EC/EC^* mice were exposed to high oxygen for 48 hours, returned to room air for 12 or 24 hours, and then stained for ANG2. As expected, ANG2 expression in control mice was strongly upregulated in ECs along the sprout front adjacent to the avascular central retina at both time points (Figure 7A). As previously reported for this antibody (61, 62), ANG2 was expressed preferentially in ECs located at the sprout front, consistent with tip cell identity (Supplemental Figure 6A). In *Bak^–/–^ Bax^EC/EC^* mice we found strong ANG2 expression in the reassembling endothelium in the central retina 12 hours after return to room air (Figure 7B). By 24 hours, when most reassembly was complete, only a few patches of ECs with strong ANG2 expression remained (Figure 7B, yellow arrows).

To test whether ANG2 was necessary for vessel reassembly, *Bak^–/–^ Bax^EC/EC^* mice were exposed to 48 + 24 RA to induce network fragmentation and reassembly and treated with either an ANG2-neutralizing antibody (63) or an isotype control antibody on return to room air (Figure 7C). No difference was observed in the central retina vessel area or network fragmentation between treatment groups in the *Bak^–/–^ Bax^EC/EC^* mice, suggesting that ANG2 inhibition did not inhibit vessel reassembly (Figure 7, D–F). ANG2 inhibition did, however, reduce endothelial tip cell activity in the retina. WT mice treated with ANG2-neutralizing antibody following return to room air showed a 65% reduction in tip cells based on the morphological criteria of filopodial clusters (Supplemental Figure 6, B and C), consistent with the known role of ANG2 in promoting tip cell activity in the retina (53). Consistent with this, ANG2 inhibition prevented the formation of new vessel networks from the reassembled vessels by sprouting angiogenesis. Whereas extensive superficial- and mid-layer vasculature was present in ANG2-inhibited *Bak^–/–^ Bax^EC/EC^* retinas 5 days after return to room air as a result of vessel reassembly (Figure 7, G and H), these animals contained less deep-layer vasculature compared with isotype control–treated mice (Figure 7, G–I). Deep-layer vessels form from vessel sprouts that emerge from the superficial layer. We observed strong ANG2 expression in these sprouts in the periphery of *Bak^–/–^ Bax^EC/EC^* retinas 24 hours after return to room air (Figure 7B, pink arrows), and formation of these vessels during normal retinal development is impaired in *Ang2* mutants (55, 56). Taken together, these data suggest that ANG2 is not required for the initial process of vessel reassembly, but is required for further expansion of the reassembled vessels by sprouting angiogenesis.

## Discussion

While blocking apoptosis has previously been shown to prevent neovascularization in the OIR model (23), the way it did so was unknown. We show that protecting against BAK/BAX-dependent apoptosis did not prevent the initial loss of perfusion or breakdown of the vasculature through vessel regression that leads to areas of the retina becoming ischemic. Rather, it allowed those ECs that would ordinarily die during the process of vessel regression to survive and persist. These did not persist as intact or functional vessels, but instead as isolated clusters of cells scattered throughout the ischemic zones. As the tissue surrounding these isolated ECs became hypoxic, they underwent a sprouting angiogenic response. Through this, they reestablished connections with each other and neighboring vessels, reassembling into a functional network. These reassembled vessels rapidly restored oxygen supply to the ischemic tissue, thereby diminishing hypoxia-induced pathological responses (Supplemental Figure 6D). Our data show that this mechanism replaces vessels sooner than normal angiogenesis would, which must grow a new network (including replacement ECs lost to apoptosis during vaso-obliteration) from vessels peripheral to the ischemic lesion (Supplemental Figure 6D).

We found that ischemia in the OIR model was caused by EC apoptosis–independent vessel closure, predominantly in arterial side-branch vessels. Vessel closure in the OIR model has been shown to depend on DLL4/Notch signaling through the regulation of vasoactive gene expression (41). Arterial side-branch closure led to the loss of blood flow to downstream capillaries, suggesting that loss of blood flow shear constitutes the major initiating event in vaso-obliteration. Previous studies have suggested that vessel closure occurs before apoptosis (41, 42). When apoptosis was blocked, capillaries deprived of blood flow still initiated a vessel regression response in which vessels disassembled and ECs retracted into isolated clusters resulting in network fragmentation. The vessel regression seen in the absence of apoptosis likely involves a process of cell migration similar to that which occurs in normal angiogenic vessel pruning. That process also occurs in response to blood flow changes (25–28) and is independent of EC apoptosis (24).

When protected from apoptosis, ECs in the fragmented, nonperfused vessels retained the capacity to initiate a sprouting angiogenesis phenotype. This occurred in response to hypoxia. These cells displayed behavioral and morphological features typical of endothelial tip cells that guide the growth of new vessels during sprouting angiogenesis (64). This process is responsible for the vascularization of the retina in response to physiologic hypoxia (1, 65). Through this behavior, the isolated cells were able to reestablish connections with neighboring vessels and reassemble into a functional network. In some instances, ECs appeared to migrate along predetermined paths. Empty vascular basement membranes left behind during vessel regression can act as a scaffold for vessel regrowth (66, 67). It is likely that this was occurring during vessel reassembly, as the PECAM1/collagen IV ratio returned to normal during vessel reassembly, suggesting that it involves, at least to some extent, recanalization of empty basement membranes.

Unlike normal sprouting angiogenesis, in which EC proliferation is coupled to new-vessel growth (47), the sprouting and network reassembly we observed in the absence of apoptosis proceeded largely without the need for EC proliferation. While the exact reasons for this lack of proliferation are unclear, the unchanging number of ECs throughout the process of network fragmentation and reassembly possibly suggests some form of negative feedback mechanism on proliferation. Additionally, many of the reassembling ECs showed morphological and molecular features consistent with tip cell activity/identity, a state known to have less proliferative activity than other EC types (64). Nonetheless, the formation of extensive deeper-layer vasculature suggests that reassembled vessels retain the capacity for further network growth that likely does require EC proliferation. Deep-layer retinal vessels grow from vessel sprouts originating from the superficial vascular layer and have not yet formed when mice start the OIR procedure. These vessels therefore do not reassemble from existing EC clusters in the apoptosis-resistant mutants in the same way the superficial layer does. The presence of deep-layer vasculature in *Bak^–/–^ Bax^EC/EC^* mice 5 days after return to room air therefore suggests that reassembled superficial layer vessels are competent to undergo further sprouting angiogenesis to give rise to the new vessel networks in the deeper layers. These vessels are important for normal retina function, as defects in their formation are associated with diseases that cause vision loss (68).

Vessel reassembly occurred in response to hypoxia, prompting us to investigate whether it was dependent on the hypoxia-induced, proangiogenic growth factors VEGFA and ANG2. Both VEGFA and ANG2 are upregulated in the OIR model following the onset of hypoxia (36, 48, 57) and are required for pathological neovascular lesion formation (44, 45, 49, 69). Our data show that reducing VEGFA to levels that suppress pathological angiogenesis did not interfere with the vessel reassembly that occurs in the absence of EC apoptosis. While these findings do not necessarily exclude a role for low-level VEGFA in the reassembly process, they do show that it can proceed at levels of VEGFA that are limiting for pathological angiogenesis, or reduce ESM1 expression by 94%. While the effects of VEGFA-neutralizing antibodies on neovascularization and ESM1 expression that we observe suggest direct inhibition in the retina based on other experimental evidence (44, 45, 49, 51), we cannot rule out systemic effects following intraperitoneal administration of the neutralizing antibodies.

ANG2 is a proangiogenic growth factor expressed in endothelial tip cells (52–54). It is essential for sprouting angiogenesis in the retina (53, 55, 56). Despite the fact that ANG2 was upregulated in the ECs of reassembling vessels, neutralization of ANG2 did not prevent vessel reassembly. ANG2 was, however, required for reassembled vessels to undergo subsequent angiogenic expansion to form deep-layer vasculature. The requirement for ANG2 in the formation of deep-layer vessels has been previously demonstrated in the context of normal, developmental retinal angiogenesis (55, 56). While we cannot rule out the possibility that some level of ANG2 activity is required for vessel reassembly, our results suggest that the threshold is lower than that needed for normal sprouting angiogenesis. While the effects of ANG2 neutralization on tip cell activity, sprouting angiogenesis, and deep-layer vessel formation match previous genetic and inhibitor-based studies in vivo (53, 55, 56) and in vitro (70, 71) and suggest direct neutralization of ANG2 in the retina, we cannot rule out systemic effects following intraperitoneal administration of the neutralizing antibodies. We also cannot rule out the possibility that vessel reassembly occurs before the full effect of ANG2- or VEGFA-neutralizing antibodies manifests. However, vessel reassembly became less efficient following longer exposure to high oxygen, and VEGFA and ANG2 neutralization still did not prevent reassembly even following longer (3 days) exposure to high oxygen.

Sprouting angiogenesis and tip cell activity are regulated by many signaling inputs in addition to VEGFA and ANG2. These include BMPs, FGFs, Notch, cell adhesion, and direct hypoxia sensing, among others (72), any of which may be essential during the vessel reassembly process. In addition to forming new vessel sprouts indicative of tip cell activity, vessel reassembly also appeared to involve recanalization of preexisting basement membranes. This process may not be as dependent on factors involved in normal sprouting angiogenesis and may point to the requirement of other pathways, the identity of which will require further investigation.

The reassembly of vessel fragments we observed bears similarities to vessel formation in other contexts. During vasculogenesis, angioblasts coalesce into endothelial cords before establishing lumenized vessels (2); isolated clusters of lymphatic ECs incorporate into growing lymphatic vessel networks (73); and isolated vessel segments in the rat mesentery reconnect during network growth (74). We showed that isolated ECs protected from apoptosis driven by BAK and BAX can respond to hypoxic stimuli to reassemble themselves into a functional vessel network, resulting in rapid network repair and tissue reoxygenation. This had the effect of reducing hypoxia, pathological neovascularization, and reactive retinal gliosis. Manipulation of other pathways, such as ANG1 (44) and ATM signaling (75), has also been shown to accelerate the repair of the retinal network and reduce neovascularization in the OIR model. In these cases, vaso-obliteration proceeded normally, and enhanced centripetal growth of the peripheral vascular network accounted for the vessel repair and took longer than the reassembly process we describe (44, 75). Blocking apoptosis in the OIR model therefore enabled a fundamentally different and faster approach for restoring blood supply to the ischemic retina.

Network reassembly became less efficient the longer ECs were exposed to high oxygen and therefore in the fragmented, flow-deprived state. While this reduced efficiency may simply reflect the fact that more fragmentation had occurred at these later time points, taking longer to repair, other factors may contribute to hinder the process. By 48 hours, ECs had downregulated the transcription factor FLI1 and may downregulate other genes that may affect their responsiveness to angiogenic stimuli. Changes to the neurovascular unit over time likely also affect reassembly. Astrocytes promote retinal angiogenesis both in normal development (37) and in vessel regrowth in the OIR model (44, 76). The astrocyte network deteriorates rapidly beyond 24 hours of exposure to high oxygen (77). This may explain why revascularization in our model was less efficient, but still effective, beyond 24 hours of high oxygen exposure.

Current treatments for neovascular disease in the retina revolve around reducing the angiogenic stimulus. This is done either by decreasing the metabolic demand of the retina or by directly inhibiting VEGFA (8–12). Our findings show that in the OIR model for retinopathy of prematurity, preventing EC apoptosis can accelerate revascularization of the retina and reduce the hypoxic stimulus that drives abnormal VEGFA expression. Our data show that protecting ECs from apoptosis in this model enables them to persist within ischemic tissue without the need for ongoing blood flow support and reestablish functional vessels sooner than normal angiogenic growth can achieve. Furthermore, like VEGFA inhibition, blocking apoptosis in the OIR model was effective at preventing subsequent neovascular response. These findings may also have implications for restoring blood flow in other ischemic retinopathies and diseases such as stroke and myocardial infarction.

## Methods

### Mice.

Conditional *Bax* mice (78), *Bak-*null mice (79), *Tie2-Cre* mice (80), *Cdh5(PAC)-CreERT2* mice (32), *caspase-8–*null mice (81), *Mlkl-*null mice (82), and *ROSA26^Sortm4(ACTB-tdTomato,-EGFP)Luo^* (*mTmG*) mice (46) have been previously described. Conditional *Vegfr2* mice crossed with *Tie2-CreERT2* have been described previously (39) and show 80% reduction of endothelial VEGFR2 protein along with loss of ECs from nonretinal organs following tamoxifen administration (39, 83). Animals were maintained on an inbred C57BL/6 background. The day of birth was termed P0. Mice of both sexes were used. *Bak^–/–^ Bax^iEC/iEC^* mice were injected with 50 μg tamoxifen (MP Biomedicals; dissolved in sterile corn oil plus 5% ethanol) by intragastric injection at P2 and P3. For conditional *Vegfr2* mice, tamoxifen (MilliporeSigma) was dissolved in ethanol and diluted in sterile corn oil to 1 μg/μL, mice were injected i.p. with 50 μg at P7, P8, and P9, and eyes were dissected for analysis at P10. Control genotypes for experiments involving *Bak^–/–^ Bax^EC/EC^* and *Bak^–/–^ Bax^iEC/iEC^* mice were *Bak^–/–^ Bax^fl/+^ Cre^+^*, *Bak^–/–^ Bax^fl/+^ Cre^–^*, and *Bak^–/–^ Bax^fl/fl^ Cre^–^*.

### Oxygen-induced retinopathy.

Nursing dams with P7 pups were housed in a Perspex chamber (BioSpherix) and exposed continuously to 74% ± 1% oxygen in air maintained by a ProOx110 oxygen controller (BioSpherix). Duration of oxygen exposure and subsequent recovery time in room air is indicated in each figure. Pups were fostered to BALB/c females following exposure to 3 days of high oxygen to prevent oxygen toxicity in dams.

### VEGFA and ANG2 neutralization experiments.

Mouse anti–mouse/human VEGFA neutralizing antibody B20-4.1.1 (50) (Genentech) and control mouse anti–human CD8a antibody OKT8 (WEHI Antibody Facility) were administered to mice by i.p. injection at the time points indicated either at 5 mg/kg or at the dose stated in the figure legend. Human anti–mouse/human ANG2 antibody ABA (63) (provided by Gou Young Koh, KAIST, Daejeon, South Korea) and control human anti–respiratory syncytial virus antibody palivizumab (provided by Steven A. Stacker, Peter MacCallum Cancer Centre, Melbourne, Victoria, Australia) were administered to mice by i.p. injection at 20 mg/kg at the time points indicated.

### Immunohistochemical staining.

For whole-mount immunohistochemistry, eyes were fixed for 2 hours in 4% paraformaldehyde at 4°C before dissection and blocking of retinas for 1 hour at room temperature in Dulbecco’s phosphate-buffered saline (DPBS) with 1% Triton X-100 and 2% donkey or goat serum. Retinas were stained with primary antibodies prepared in blocking solution overnight at 4°C, washed in DPBS containing 0.01% Triton X-100, then stained overnight with secondary antibodies prepared in blocking solution. Primary antibodies were rat anti-PECAM1/CD31 (BD Pharmingen, 553370, clone MEC13.3), goat anti-PECAM1/CD31 (R&D Systems, AF3628), goat anti–collagen IV (Merck, AB769), rabbit anti–cleaved (active) caspase-3 (Cell Signaling, 9664, clone 5AE1), rat anti-ICAM2/CD102 [BD Pharmingen, 553326, clone 3C4(mIC2/4)], rat anti–VE cadherin (BD Pharmingen, 555289, clone 11D4.1), rabbit anti-pimonidazole (Hypoxyprobe, PAb2627AP), rabbit anti-NG2 (Merck, AB5320), rabbit anti-ERG (Abcam, ab110639, clone EPR3863), rabbit anti-FLI1 (Abcam, ab15289), rabbit anti-GFAP (DAKO, Z0334), human anti-ANG2 (4H10) (63), rabbit anti–phospho–histone H3 (Ser10) conjugated to AF488 (Merck, 06-570-AF488), and goat anti-ESM1 (R&D Systems, AF1999). Secondary antibodies were donkey anti-rabbit–Cy3 (711-165-152), donkey anti-rabbit–AF647 (711-605-152), donkey anti-rat–DL488 (712-485-153), donkey anti-rat–Cy3 (712-165-150), donkey anti-rat–AF647 (712-605-150), donkey anti-goat–DL405 (705-475-147), donkey anti-goat–Cy3 (705-165-147), and streptavidin-AF488 (016-540-084) (all from Jackson ImmunoResearch Laboratories). For isolectin B4 (Vector Laboratories, B-1205) staining, retinas were blocked in 1% BSA, 0.3% Triton X-100 in DPBS, then incubated with isolectin B4 in 0.4% Triton X-100 in HBSS. Retinas were mounted with Prolong Diamond (Invitrogen, P36961). For hypoxia detection, pups were injected i.p. with 60 mg/kg pimonidazole (Hypoxyprobe) either immediately after exiting the oxygen chamber (30 minutes labeling duration) or 24 hours after exiting the chamber (2 hours labeling duration). Pimonidazole was detected using rabbit anti-pimonidazole antibody and staining performed as above. For i.v. lectin perfusion, P7 pups were anesthetized using Xylazil-20 (20 mg/kg; Troy Laboratories Pty. Ltd.) and ketamine (100 mg/kg; Hospira Australia Pty. Ltd.) via i.p. injection; then 30 μL of DyLight488-conjugated *Lycopersicon esculentum* lectin (Vector Laboratories, DL-1174) was injected retro-orbitally and allowed to circulate for 2 minutes before retinas were fixed, dissected, and stained as above. In mice subjected to 48 + 24 RA, *L*. *esculentum* lectin was injected intracardially, via the left ventricle. For cryosectioning, P18 retinas were fixed for 24 hours, equilibrated in 30% sucrose in DPBS for at least 1 hour at room temperature, and frozen in OCT embedding compound (Scigen). Twenty-micrometer cryosections were adhered to Polysine slides (Thermo Scientific), washed in DPBS, then blocked and stained as for whole-mount retinas.

### Imaging and image analysis.

Retinas were imaged with a Leica TCS SP8 confocal microscope using ×10/0.4 NA, ×20/0.75 NA, or ×40/1.30 NA objectives and Leica Application Suite software. All image analysis was performed in the Fiji distribution of ImageJ software (NIH) (84). Apoptotic ECs (defined as cleaved [active] caspase-3^+^/PECAM1^+^ cells enclosed by collagen IV signal) were quantified manually from confocal *Z*-stack images from the central retina at P8 and normalized to central retina area. Central retina vessel area was calculated based on PECAM1 signal from maximum-intensity projection images following application of a median filter (2 pixels) and a “despeckle” filter before manual adjustment of threshold and measurement of area. Vessel area was normalized to total area of the central retina. Vessel regression was determined from equivalent areas as a ratio of PECAM1^+^ vessel segment length to collagen IV^+^ vessel segment length in a semiautomated fashion. Binary masks of both PECAM1 and collagen IV channel were made manually by various morphological filters and thresholding signal. Collagen IV^+^ PECAM1^–^ vessel segment mask (i.e., regressing vessels) was generated by subtraction of PECAM1 mask from collagen IV mask. Collagen IV^+^ PECAM1^–^ mask and collagen IV mask were then skeletonized, and length of vessels within each mask was measured. Collagen IV^+^ PECAM1^–^/collagen IV ratio was generated automatically based on vessel length. Data are represented as PECAM1/collagen IV ratio. ICAM2/collagen IV ratio was calculated in the same way, replacing PECAM1 with ICAM2 signal. Arterial side branches were counted as perfused if lectin signal overlapped continuously with ICAM2. Number of perfused arterial side branches within the central retina was counted and normalized to artery length. Nonperfused side branches were further categorized as “attached,” “disrupted,” or “detached” based on ICAM2 signal. “Attached” vessels were not perfused but had normal ICAM2 signal (identical appearance to perfused side branches). “Disrupted” side branches had abnormal ICAM2 morphology compared with normal vessels, suggesting they were in the process of closing their lumen and detaching from the artery. “Detached” side branches did not have continuous ICAM2 signal between side branch and major artery but did have continuous collagen IV signal. EC number was counted within equivalent regions of the same size and normalized to retina area. EC nuclei were counted manually based on ERG or FLI1 nuclei in cells positive for vascular markers (isolectin B4 or PECAM1). Number of FLI1 particles (nuclei) was counted after application of a median filter (2 pixels), removal of noise with “despeckle” function, and analysis of particles greater than 20 square pixels. Particles were then manually checked through confocal *Z*-stacks to ensure all ECs were counted. EC proliferation was determined by manual counting of phospho–histone H3 (Ser10)^+^ FLI1^+^ nuclei and normalized to total EC number in each region of the retina. ECs were then assigned as peripheral or central region based on demarcation described above. Network fragmentation, vessel branch points, and vessel width were quantified from PECAM1 images of central retina vasculature. A median filter (2 pixels) was first applied to the PECAM1 images, which were then segmented using the Trainable Weka Segmentation Plugin in Fiji (85). Segmented images were then skeletonized. The number of separate, independent network skeletons was used as a measure of network fragmentation. The number of skeletons and the number of vessel branch points were both normalized to central retina area. Vessel width was calculated as total vessel area divided by total vessel length calculated from Weka-segmented images. Hypoxic area in the central retina was quantified from pimonidazole images. A 20-pixel median filter was applied to the images and a signal intensity threshold used to distinguish hypoxic signal from background. Hypoxic area was normalized to total central retina area. Neovascular tufts were quantified based on maximum-intensity projection images of collagen IV signals, with neovascular lesions defined as bright, globular collagen IV^+^ structures. These were segmented manually in Adobe Photoshop CC 2015 and the area calculated in Fiji. Neovascular area per retina was normalized to total retina area. Where necessary, a despeckle filter was applied to select channels in images displayed in figures for clarity.

### Retina live imaging.

*Bak^–/–^ Bax^iEC/iEC^ mTmG* mice were injected with tamoxifen as described above by intragastric injection at P2 and P3. Dam and pups were exposed to high oxygen for 48 hours followed by 12 hours in room air. Retinas were then dissected immediately in cold DMEM, and 5 radial incisions were made; then retinas were flat-mounted with the internal limiting membrane face-down on a disc of 1% low-melting-point agarose gel dissolved in DMEM containing 10% FCS, and set in a 35-mm culture dish with coverglass bottom (Eppendorf, 0030740017). Nitex 50-μm filter mesh (Sefar, 03-50/31) dipped in molten 1% agarose/DMEM/10% FCS mixture was laid on top of the retina and allowed to set briefly to minimize retina movement during imaging. The dish was then fixed into a custom 3D printed stage insert with inbuilt water reservoirs to maintain humidity. Retinas were live-imaged with an inverted Leica SP8 confocal microscope using a HyD detector and a ×10/0.4 NA objective. Retinas were maintained at 37°C and 5% humidified CO_2_. Images were acquired every 30 minutes for 5 hours. After acquisition, a mean filter (1 pixel) was applied to the images using Fiji software.

### VEGFA ELISA.

VEGFA levels in whole retina lysates were quantified using a mouse VEGF Quantikine ELISA kit (R&D Systems, MMV00) per the manufacturer’s instructions. Briefly, freshly dissected whole retinas were snap-frozen in dry ice, thawed in 50 μL of DPBS, and homogenized by manual trituration 20 times. Retina homogenates were subject to 2 freeze/thaw cycles, then centrifuged at 5000 *g* for 5 minutes. An equal volume of lysate was then assayed per retina. VEGFA concentrations were calculated from a standard curve generated by 4-parameter logistic regression analysis in R version 3.4.4 using the drc package (86).

### Statistics.

All data are shown as mean ± SEM. Statistical analyses were performed for all quantitative data using Prism 7.0 (GraphPad) or in R version 3.4.4 where specified. *P* values below 0.05 were considered significant.

### Study approval.

All experiments involving animals were performed with procedures approved by The Walter and Eliza Hall Institute of Medical Research Animal Ethics Committee or with University of Nottingham Animal Welfare and Ethical Review Board approval.

## Author contributions

ZLG performed experiments, analyzed data, and wrote the manuscript. LW developed analytical tools and analyzed data. VHYW, ZH, and BVB performed experiments and analyzed data. RYY, ARM, and AVB performed experiments. DOB analyzed data. KB and CP advised on live-imaging protocol. RCAS provided essential equipment and conceptual advice and analyzed data. LC conceived the project, performed experiments, analyzed data, and wrote the manuscript. All authors contributed to and approved the manuscript.

## Supplementary Material

Supplemental data

Supplemental Video 1

Supplemental Video 2

Supplemental Video 3

## Version 1 05/19/2020

In-Press Preview

## Version 2 07/13/2020

Electronic publication

## Version 3 08/03/2020

Print issue publication
