# Supplementary material for: Blocking endothelial apoptosis revascularizes the retina in a model of ischemic retinopathy
Source: J Clin Invest. 2020 Jul 13;130(8):4235–51. doi: 10.1172/JCI127668 (PMC7410052; doi:10.1172/JCI127668)
Supplement: Supplemental data [file jci-130-127668-s345.pdf]

Supplementary Figure 1.

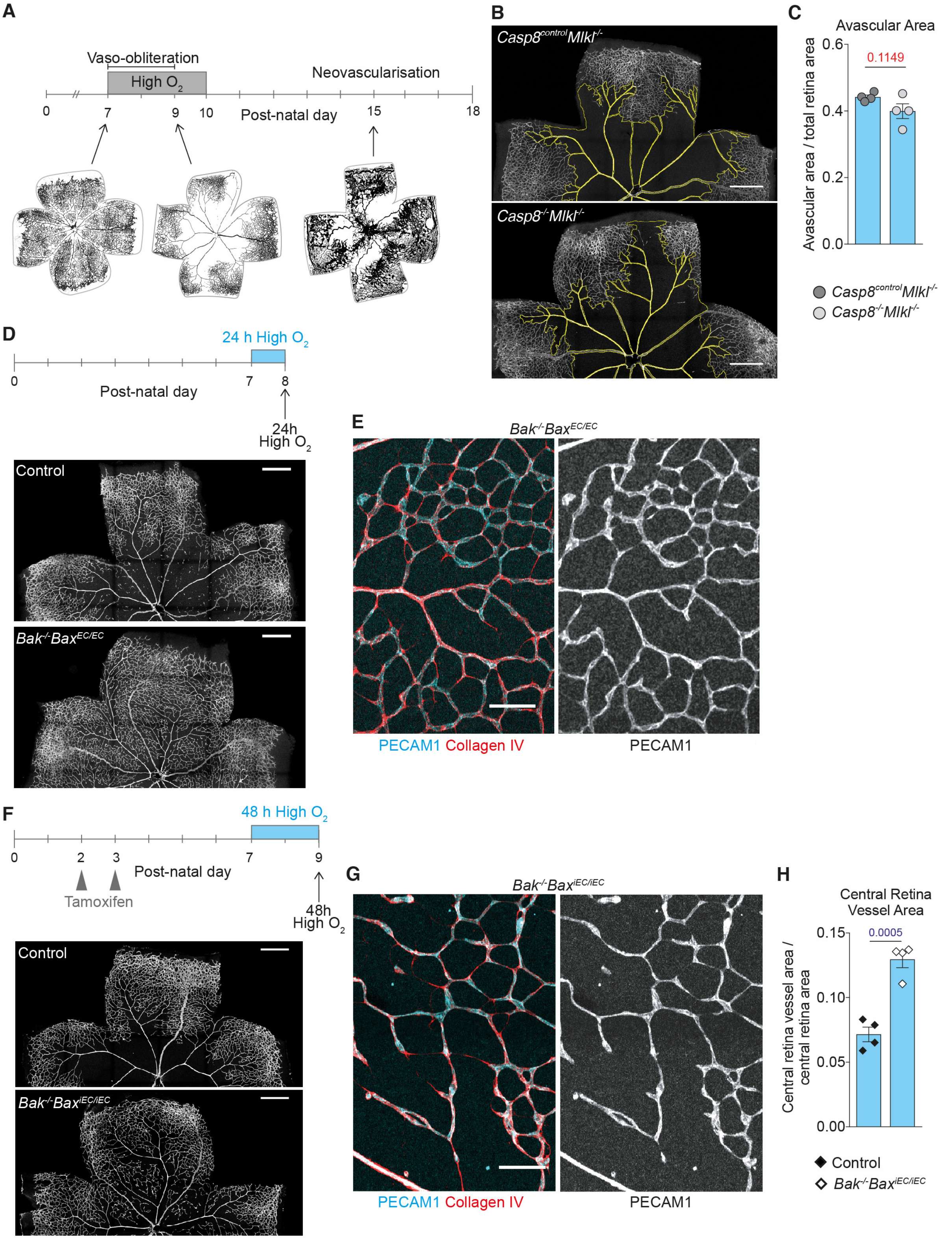

Supplementary Figure 1. (**A**) Experimental overview of entire oxygen-induced retinopathy procedure (**B**) PECAM1 staining control and *Caspase* $8^{-/-}$*Mlkl*$^{-/-}$ mice after 48 h high oxygen. Yellow lines demarcate avascular from vascularised regions. Scale bar = 500 µm. (**C**) Avascular area normalised to total retina area for control (n = 4) and *Caspase* $8^{-/-}$*Mlkl*$^{-/-}$ (n = 4)

mice after 48 h high oxygen. Student's two-tailed t-test. (**D**) Experimental overview of mice subjected to 24 h high oxygen. PECAM1 stained retinas of control and *Bak*$^{-/-}$*Bax*$^{EC/EC}$ mice after 24h high oxygen, scale bar = 500 µm. (**E**) Collagen IV (red) and PECAM1 (cyan) staining in 24 h high oxygen *Bak*$^{-/-}$*Bax*$^{EC/EC}$ central retina. Scale bar = 80 µm. (**F**) Experimental overview of mice treated with tamoxifen and subjected to 48 h high oxygen. PECAM1 stained retinas of control and *Bak*$^{-/-}$*Bax*$^{iEC/iEC}$ mice after 48 h high oxygen, scale bar = 500 µm. Deletion was induced by administering tamoxifen at P2 and P3. (**G**) Collagen IV (red) and PECAM1 (cyan) staining within *Bak*$^{-/-}$*Bax*$^{iEC/iEC}$ central retina. Scale bar = 80 µm. (**H**) Central retina vessel area normalised to central retina area in control (n = 4) and *Bak*$^{-/-}$*Bax*$^{iEC/iEC}$ (n = 4) mice after 48 h high oxygen. Student's two-tailed t-test. All data are mean ± SEM. Each circle represents one animal.

Supplementary Figure 2

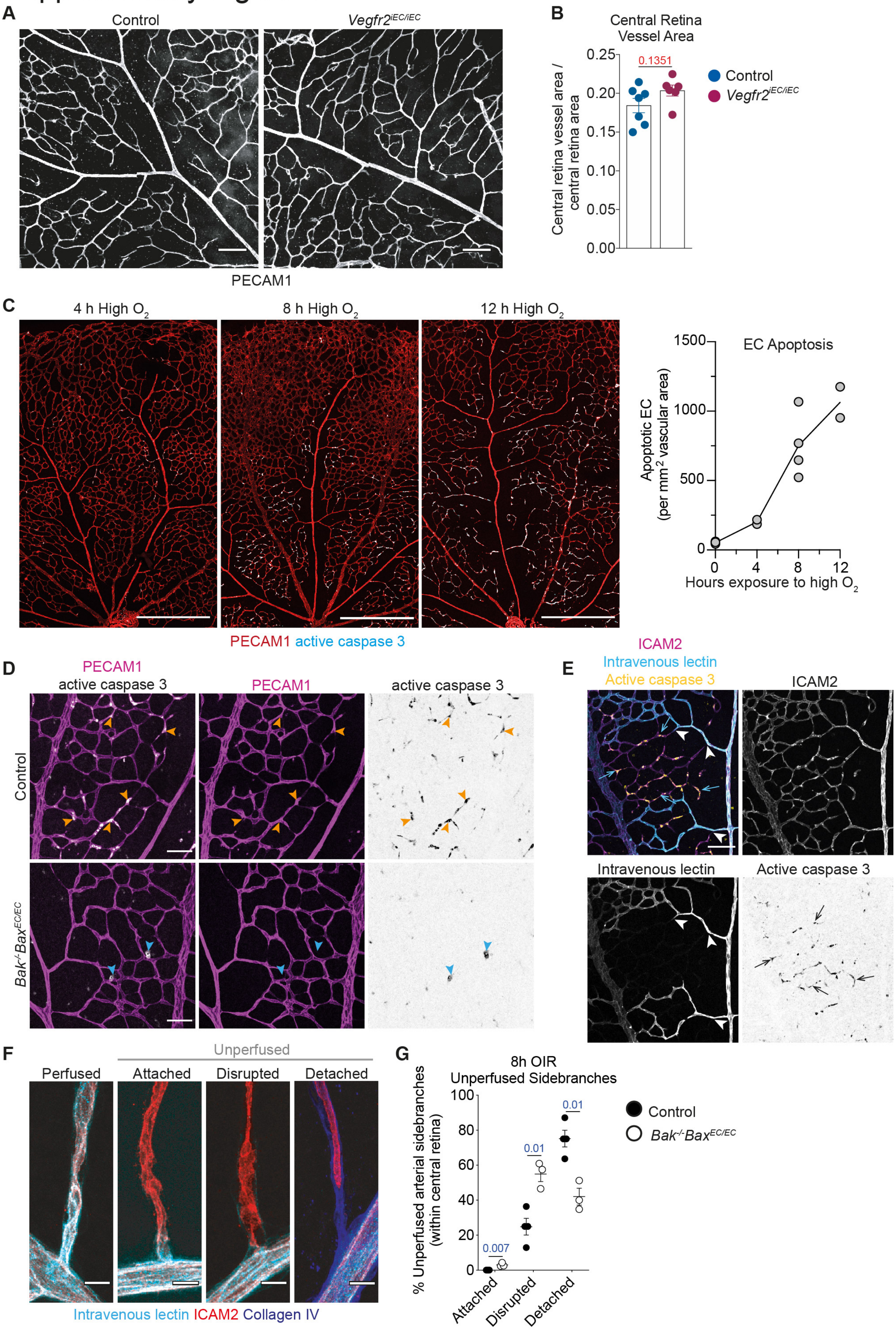

Supplementary Figure 2. (**A**) PECAM1 staining of P10 control and *Vegfr2*$^{iEC/iEC}$ central retinas. Scale bar = 100 µm. (**B**) Quantification of P10 central retina vessel area normalised to central retina area in control (n = 7) and *Vegfr2*$^{iEC/iEC}$ mice (n = 6). Student's two-tailed t-test. (**C**) PECAM1 (red) and active caspase 3 (cyan) stained images of P7 wild type retinas after 4, 8 and 12 h high oxygen and quantification of apoptotic ECs at each time point (0 h n = 2, 4 h n = 2, 8 h n = 2, 12 h n = 2). Scale bar = 500 µm. Line indicates mean. Each circle represents one animal. A PECAM1 mask was applied to the active caspase 3 signal to exclude non-EC apoptosis. (**D**) Control and *Bak*$^{-/-}$*Bax*$^{EC/EC}$ retinas following exposure to high oxygen for 8 h stained for PECAM1 (magenta) and active caspase 3 (grey). Orange arrowheads indicate examples of EC apoptosis, blue arrowheads indicate non-EC apoptosis. Scale bar = 50 µm. (**E**) Control retina intravenously perfused with lectin (cyan) and stained for ICAM2 (magenta) and active caspase 3 (yellow). Arrowheads indicate representative down-stream vessel closure points. Scale bar = 80 µm. (**F & G**) Representative examples and quantification of perfused (lectin+, cyan) and types of unperfused arterial side branches in retinas from control (n = 4) and *Bak*$^{-/-}$*Bax*$^{EC/EC}$ mice (n = 3) co-stained for ICAM2 (red) and collagen IV (blue). Scale bar = 10 µm. Multiple t-tests using Holm-Sidak correction for multiple comparisons. All data are mean ± SEM. Each circle represents one animal.

Supplementary Figure 3.

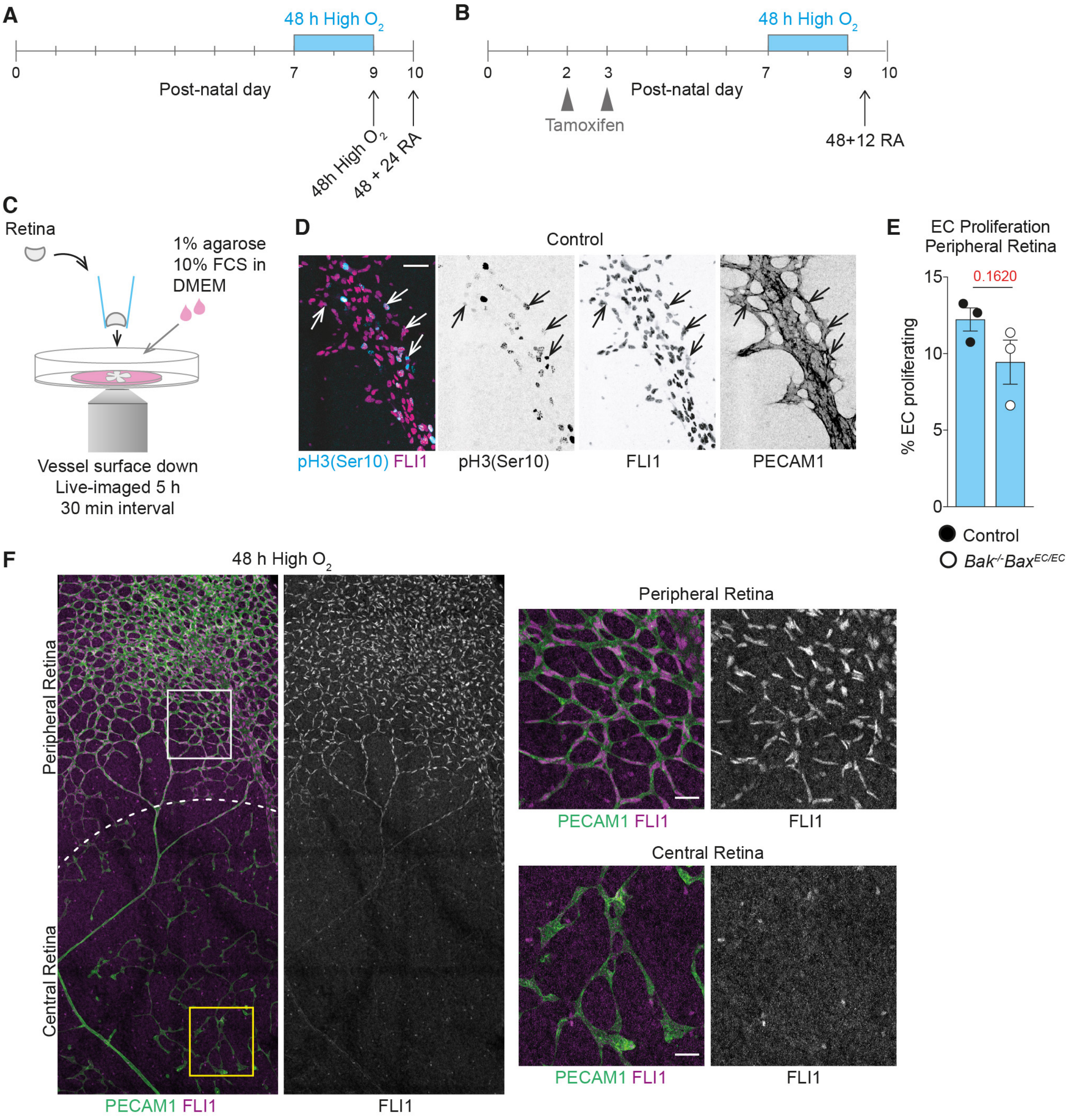

Supplementary Figure 3. (**A**) Experimental overview of mice subjected to 48 h high oxygen or 48 h high oxygen followed by a further 24 h in room air. (**B**) Experimental overview of *Bak*$^{-/-}$*Bax*$^{iEC/iEC}$*mTmG*$^{ki/+}$ mice treated with tamoxifen and subjected to 48 h high oxygen followed by 12 h in room air (48 + 12 RA). (**C**) Schematic diagram of *ex vivo* retina live imaging setup. (**D**) 48 + 24 RA control retina stained for FLI1 (magenta) and pH3(Ser10) (cyan) to visualise proliferating EC. Also stained with PECAM1. Arrows point to proliferating EC in "plexus" region. Scale bar = 50 µm. (**E**) Proportion of ECs proliferating within peripheral retina of 48 + 24 RA control (n = 3) and *Bak*$^{-/-}$*Bax*$^{EC/EC}$ (n = 3) mice. Student's two-tailed t-test. Data are mean ± SEM. Each circle represents one animal. (**F**) FLI1 (magenta and grey) expression in *Bak*$^{-/-}$*Bax*$^{EC/EC}$ retina following exposure to high oxygen for 48 h. Also stained with PECAM1 (green). White and yellow boxes indicate enlarged images of peripheral and central retina shown on right hand side. Scale bar = 30 µm.

Supplementary Figure 4.

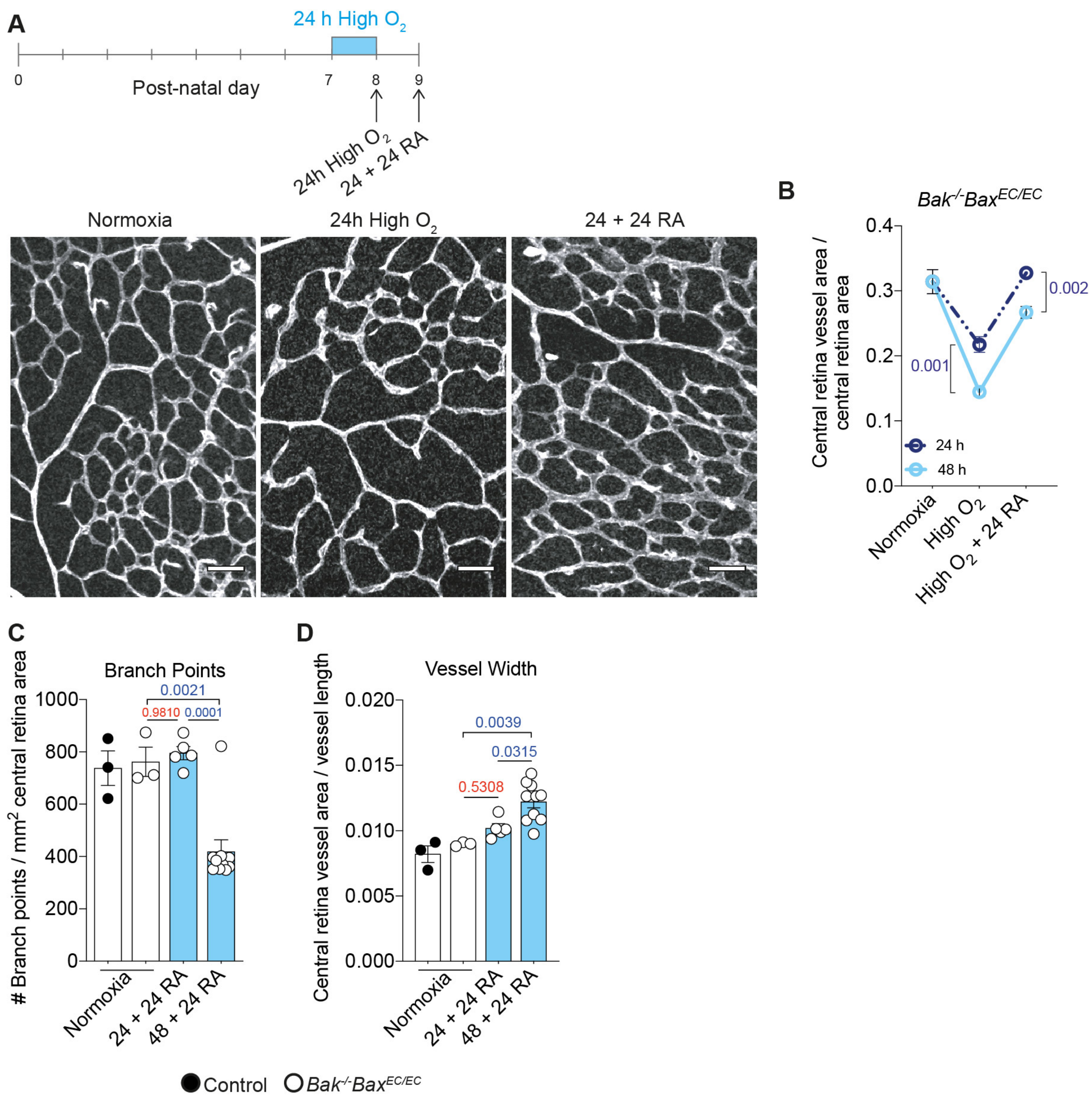

Supplementary Figure 4. (**A**) Experimental overview of mice subjected to 24 h high oxygen or 24 h high oxygen followed by a further 24 h in room air. PECAM1 stained P8 normoxic, 24 h high oxygen and 24 + 24 RA *Bak*$^{-/-}$*Bax*$^{EC/EC}$ retinas. Scale bar = 50 µm. (**B**) Comparison of central retina vessel area between mice exposed to high oxygen for 24 h or 48 h. Multiple t-tests using Holm-Sidak correction for multiple comparisons. (**C**) Branch points per mm^2 of vessel area in P8 normoxic mice (control n = 3, *Bak*$^{-/-}$*Bax*$^{EC/EC}$ n = 3) and 24 + 24 RA (n = 5) and 48 + 24 RA (n = 10) *Bak*$^{-/-}$*Bax*$^{EC/EC}$ retinas. Control retinas are devoid of capillaries after exposure to hyperoxia and hence not shown. One-way ANOVA with Tukey's multiple comparisons test. (**D**) Vessel width in P8 normoxic mice (control n = 3, *Bak*$^{-/-}$*Bax*$^{EC/EC}$ n = 3) and 24 + 24 RA (n = 5) and 48 + 24 RA (n = 10) *Bak*$^{-/-}$*Bax*$^{EC/EC}$ retinas. One-way ANOVA with Tukey's multiple comparisons test. All data are mean ± SEM. Each circle represents one animal.

Supplementary Figure 5

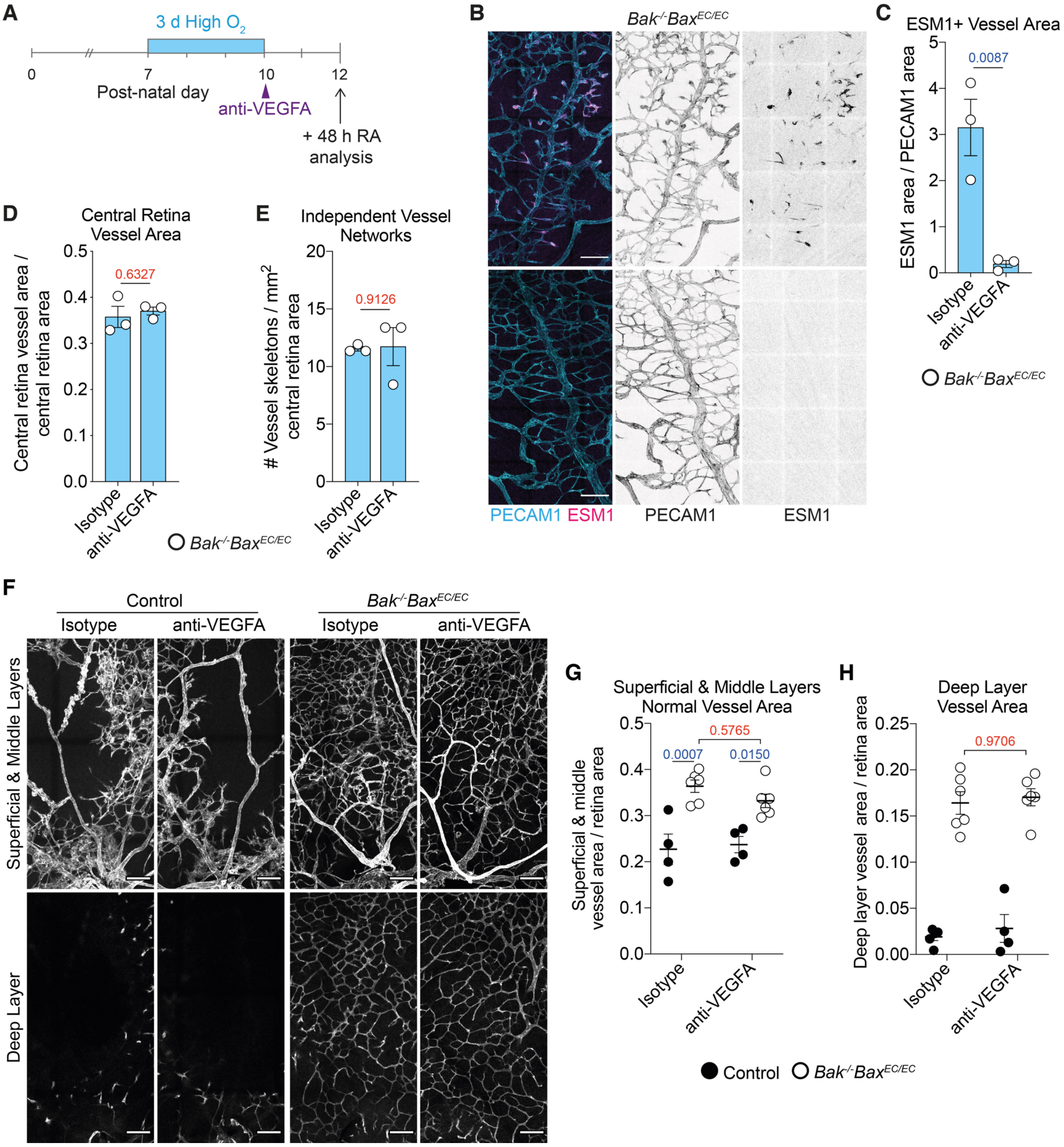

Supplementary Figure 5. (**A**) Experimental overview of mice analysed in *B – E.* (**B** and **C**) Representative images and quantification of central retinal vasculature in *Bak*$^{-/-}$*Bax*$^{EC/EC}$ mice subjected to 3 d high oxygen and 48 h in room air (3 d + 48 RA) and treated with isotype control (n = 3) or anti-VEGFA (n = 3) at 10 mg/kg. Stained for PECAM1 (cyan) and ESM1 (magenta). Scale bar = 100 µm. Student's two-tailed t-test. (**D** and **E**) Central retina vessel area and network fragmentation in 3 d + 48 RA *Bak*$^{-/-}$*Bax*$^{EC/EC}$ mice subjected treated with isotype control (n = 3) or anti-VEGFA (n = 3) at 10 mg/kg. Student's two-tailed t-test. (**F – H**) Representative images and quantification of vascular area in separate layers from the same field of view of the central retinas of control (isotype control n = 4, anti-VEGFA n = 4) and *Bak*$^{-/-}$*Bax*$^{EC/EC}$ mice (isotype control n = 6, anti-VEGFA n = 6). Scale bar = 100 µm. Two-way

ANOVA with Tukey's multiple comparisons test. All data are mean ± SEM. Each circle represents one animal.

Supplementary Figure 6

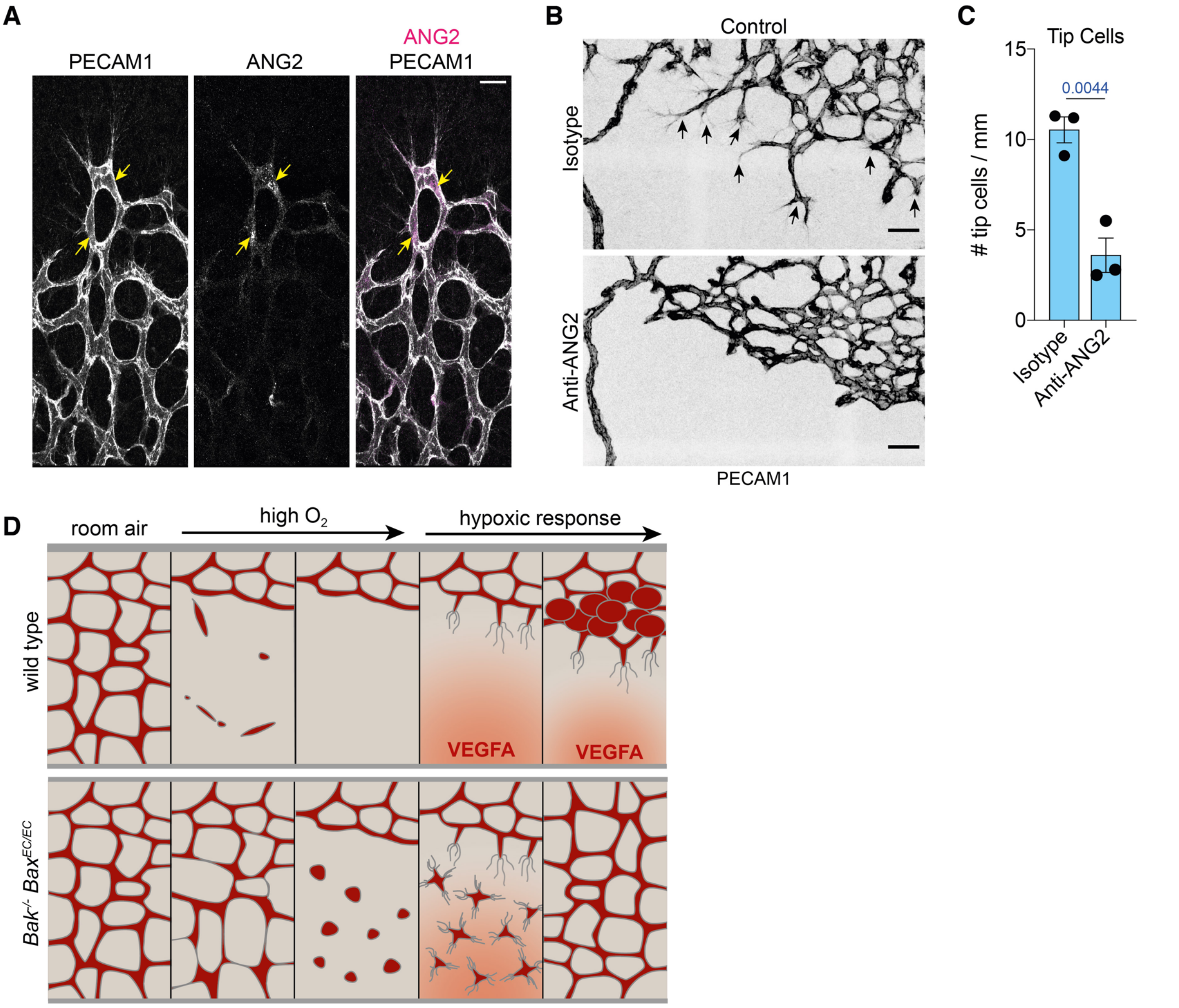

Supplementary Figure 6. (**A**) PECAM1 (grey) and ANG2 (magenta) expression in retinal tip cells (indicated by arrows) from a control mouse. Scale bar = 20 µm. (**B - C**) Images and quantification of tip cells (indicated by arrows) in control mice exposed to high oxygen for 48 h followed by 48 h RA treated with isotype (n = 3) or anti-ANG2 (n = 3). Scale bar = 50 µm. Student's two-tailed t-test. (**D**) Overview model of vessel reassembly that occurs in the absence of endothelial apoptosis in response to hypoxia-induced angiogenic factors. See text for details.
